# Selective Air Oxidation of Bis- and Trisphosphines Adsorbed on Activated Carbon Surfaces

**DOI:** 10.3390/molecules30132737

**Published:** 2025-06-25

**Authors:** Ehsan Shakeri, John C. Hoefler, Janet Blümel

**Affiliations:** Department of Chemistry, Texas A&M University, College Station, TX 77843-3012, USA; ehsan.shakeri@tamu.edu (E.S.); john.hoefler@tamu.edu (J.C.H.)

**Keywords:** phosphine oxides, air oxidation, bisphosphines, trisphosphines, activated carbon surface

## Abstract

Bis- and trisphosphines incorporating methylene and aryl spacers readily adsorb on the surface of porous activated carbon (AC). The adsorption can be performed in the absence of solvents, even when the phosphines have high melting points, or from solutions. The diverse phosphines Ph_2_PCH_2_PPh_2_ (**dppm**), Ph_2_P(CH_2_)_2_PPh_2_ (**dppe**), Ph_2_P(CH_2_)_3_PPh_2_ (**dppp**), Ph_2_P(*p*-C_6_H_4_)PPh_2_ (**dppbz**), and (Ph_2_PCH_2_)_3_CCH_3_ (**tdme**) were adsorbed in submonolayers on AC. The adsorbed phosphines were studied by ^31^P MAS (magic angle spinning) NMR spectroscopy, and their mobilities on the surface were confirmed by determining the ^31^P T_1_ relaxation times. All phosphine groups of each bis- and trisphosphine molecule are in contact with the surface, and the molecules exhibit translational mobility as one unit. All phosphines used here are air-stable. Once a submonolayer is created on the AC surface, oxygen from the air is co-adsorbed and transforms all phosphines quantitatively into phosphine oxides at room temperature. The oxidation proceeds in a consecutive manner with the oxidation of one phosphine group after another until the fully oxidized species are formed. Studies of the kinetics are based on integrating the signals in the solution ^31^P NMR spectra. High temperatures and low surface coverages increase the speed of the oxidation, while light and acid have no impact. The oxidation is fast and complete within one hour for 10% surface coverage at room temperature. In order to study the mechanism and slow down the oxidation, a higher surface coverage of 40% was applied. No unwanted P(V) side products or water adducts were observed. The clean phosphine oxides could be recovered in high yields by washing them off of the AC surface. The oxidation is based on radical activation of O_2_ on the AC surface due to delocalized electrons on the AC surface. This is corroborated by the result that AIBN-derived radicals enable the air oxidation of PPh_3_ in solution at 65 °C. When the air-stable complex (CO)_2_Ni(PPh_3_)_2_ is applied to the AC surface and exposed to the air, OPPh_3_ forms quantitatively. The new surface-assisted air oxidation of phosphines adsorbed on AC renders expensive and hazardous oxidizers obsolete and opens a synthetic pathway to the selective mono-oxidation of bis- and trisphosphines.

## 1. Introduction

The surface adsorption of molecules is immensely important for wide-ranging applications in industrial and academic settings. In contrast to covalently surface-bound species, adsorbed molecules can be removed from the surface by evaporation or washing. Therefore, adsorption is crucial for separation sciences, as well as physical and analytical chemistry [1,2,3,4,5,6].

One of the most interesting and widely used adsorbents is activated carbon (AC) [7,8,9,10,11,12,13]. The specific surface area of AC is high, and it is inexpensive and environmentally benign. Besides the ability of AC to remove air-borne pathogens and volatile organic compounds (VOCs) from the air [6,7,8], it can also function as a material that enables oxidation reactions [14,15,16,17,18,19,20,21]. For example, it has been demonstrated that sulfides [15], amines [16], NO [17,18], and benzene [19] can be oxidized on AC surfaces. At high temperatures, AC can activate molecular oxygen and allow for reactions that would otherwise need reactive oxidizers [20,21]. Recently, we communicated that after adsorbing phosphines on AC surfaces, they could be selectively oxidized at ambient temperature [22]. Different AC brands all propagate this phosphine oxidation, although the time requirements differ somewhat [22]. The air oxidation was not observed for phosphines adsorbed on silica [4], which showcases the exceptional properties of the AC surface.

When phosphines are adsorbed on silica [4] or activated carbon [22], they are attached to the surface via van der Waals interactions with their lone electron pair. Since phosphines are not covalently bound to one specific surface site, they cruise on the AC surface within the pores. The translational mobility of adsorbed phosphines has been studied by multinuclear solid-state NMR spectroscopy [4,22], which is in general a powerful analytical method for surface and materials characterization [10,12,13,23,24,25].

Phosphines are popular ligands in metal complexes, and they can even function as catalysts on their own [26]. Phosphine oxides receive less attention, but they are of growing interest because they aid in crystallization [27] and function as synthetic targets [28,29,30,31]. Phosphine oxides are also important for Mitsunobu reactions [32,33], and they enable the redox-free Mitsunobu organocatalysis [34]. Furthermore, phosphine oxides are popular surface acidity probes [35,36] because they interact strongly with various oxide surfaces like silica [37]. They can also form hydrogen bonds with diverse molecular species incorporating OH groups [38,39,40,41,42]. For example, phosphine oxides enable the creation of networks with phenols [38], and they are hydrogen-bonded to water [42,43,44]. Phosphine oxides also form strong hydrogen bonds with H_2_O_2_ and di(hydroperoxy)alkanes and stabilize these peroxides as Hilliard adducts [43,44], (R_3_PO∙H_2_O_2_)_2_, and Ahn adducts, R_3_PO∙(HOO)_2_CR’R” (R, R’ = alkyl, aryl; R” = H, alkyl, aryl) [45].

**Figure 1:**
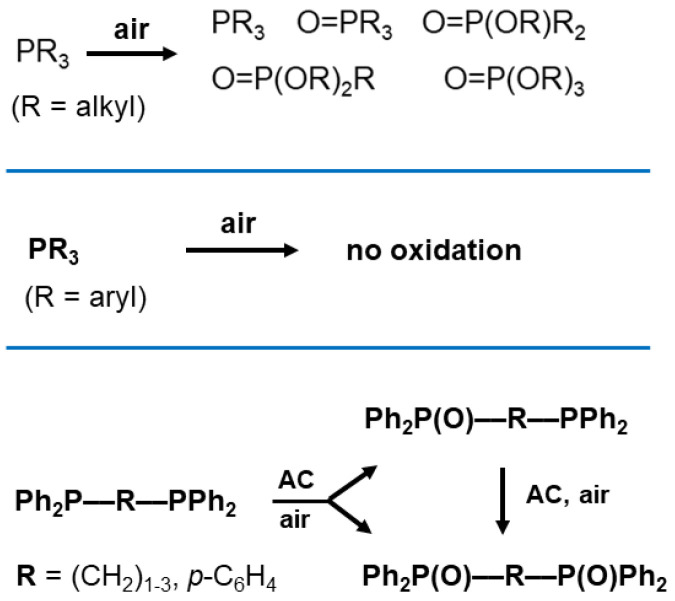
The exposure of dissolved or neat alkylphosphines to air leads to various side products (top), while triarylphosphines cannot be oxidized with air (middle) [22,43,46]. Adsorbing bisphosphines with diverse spacers **R** on activated carbon (AC) prior to air exposure results in monoxides as intermediates and dioxides as final products (bottom).

Although phosphine oxides are of growing interest, their synthesis is far from trivial [43,46,47,48,49,50]. Trialkylphosphines (PR_3_) are oxidized to a variety of inseparable P(V) species when exposed to air (Figure 1) [22,43,46]. Triarylphosphines cannot be oxidized in air, as investigated and rationalized by Buchwald [46]. Until recently, the optimal way to synthesize phosphine oxides was to treat the corresponding phosphines with aqueous H_2_O_2_ [43]. Unfortunately, this approach requires the destruction of the formed H_2_O_2_ adducts and drying the product with molecular sieves in an additional step [43].

**Figure 2:**
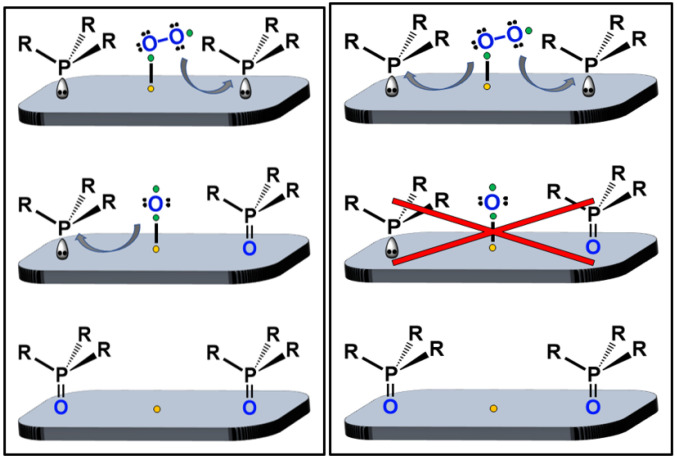
Selective oxidation of a monophosphine with air after adsorption on AC [22]. Consecutive (left) versus simultaneous (right) consumption of the O_2_ that would exclude an activated oxygen atom residing on the surface (red cross), cannot be distinguished.

Recently, we discovered that phosphines could be adsorbed on AC and oxidized in air at room temperature. The pure phosphine oxides were obtained in high yields [22]. However, this study was limited to monophosphines like P*^n^*Bu_3_ or PPh_3_ [22]. Therefore, one key question remains to be answered: Does the oxidation proceed in a consecutive or simultaneous manner (Figure 2)? Experiments with monophosphines cannot distinguish between the scenarios displayed in Figure 2. However, the difference is that in the consecutive pathways, an activated oxygen atom is generated that may reside on the AC surface as an intermediate (Figure 2, left), but not in the simultaneous oxidation of two phosphines (Figure 2, right). This difference is important because the intermediate oxygen atom has the potential to oxidize another species co-adsorbed on the surface and may enable other oxidation reactions on the AC surface under milder conditions [20,21].

**Figure 3:**
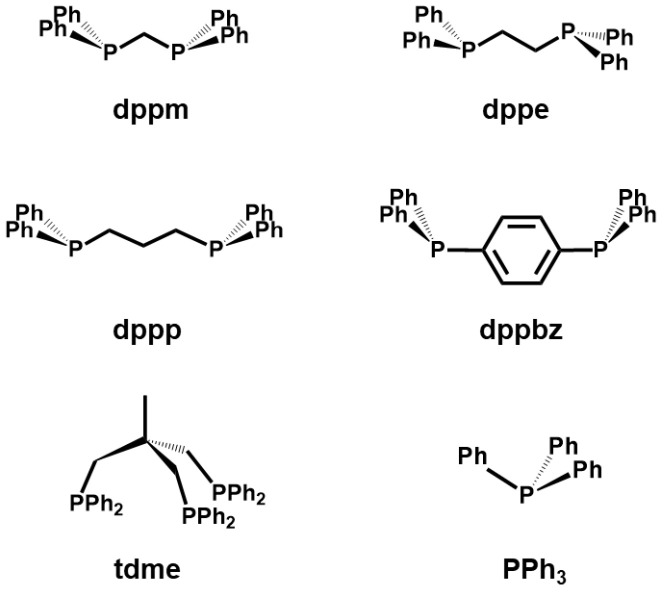
Phosphines used in this contribution to study the oxidation to the corresponding phosphine oxides after adsorption on AC.

In this contribution, we investigate the mechanism of the phosphine oxidation by using diverse bis- and trisphosphines (Figure 3). Monitoring their oxidation will allow us to distinguish between consecutive and simultaneous oxidation (Figure 2) by checking whether monoxides appear as intermediates (Figure 1, bottom).

## 2. Results and Discussion

### 2.1. Solid-State NMR of Adsorbed and Oxidized Bis- and Trisphosphines on AC Surfaces

We have communicated earlier that phosphines can be adsorbed on the surfaces within the pores of silica [4] and AC [22] even in the absence of solvents. The adsorption process does not involve the melting of the phosphines, as even PCy_3_ and PPh_3_ readily adsorb within minutes when the components are mixed manually using a mortar and pestle. Phosphines like PPh_3_ that are not air-sensitive, can be exposed to the atmosphere, and they will not be oxidized when they are adsorbed on silica surfaces [4]. Adsorbing PPh_3_ on AC, however, leads to the quantitative oxidation to OPPh_3_ [22]. It has been demonstrated for diverse monophosphines that the adsorption/oxidation procedure is widely applicable [22].

In this contribution, we demonstrate that different bis- and trisphosphines follow the same adsorption/oxidation sequence when they are applied to AC surfaces (Figure 3). Using molecules containing two or three phosphorus atoms can also provide deeper insights into the oxidation mechanism on the AC surface. Initially, the most fundamental questions are whether the adsorption of molecules with multiple phosphine groups results in translational mobility and whether the oxidation proceeds to completion, as in the cases of monophosphines.

The melting points of all bis- and trisphosphines used for this study (Figure 3) are high (**dppm** 118–119 °C; **dppe** 137–142 °C; **dppp** 63–65 °C; **dppbz** 166–170 °C; **tdme** 99–102 °C; PPh_3_ 79–81 °C). Therefore, any melting of the phosphines when they spread out on the AC surface can be ruled out. All phosphines can be adsorbed on AC by dry grinding, similar to metallocenes adsorbed on activated carbon (AC) [12]. However, to speed up the process, we applied them to the surface from solution, as described below. The surface coverages have been determined as outlined earlier [22], and the values for full monolayers that correspond to 100% surface coverage are summarized in Table S1.

**Figure 4:**
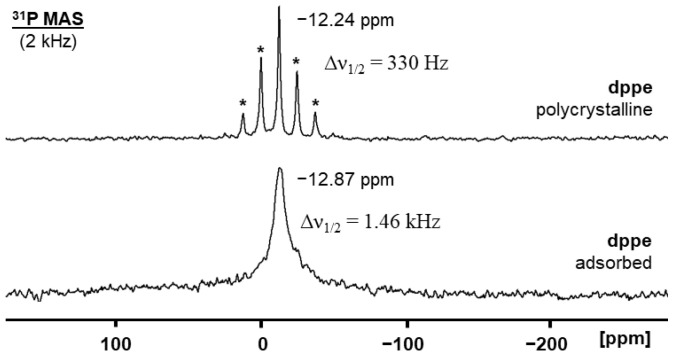
^31^P MAS NMR spectrum of polycrystalline **dppe** (top) and **dppe** adsorbed on AC with 40% surface coverage (bottom). The asterisks denote rotational sidebands.

When the representative bisphosphine **dppe** is adsorbed on the AC surface under an inert atmosphere, the ^31^P solid-state NMR characteristics change (Figure 4). Polycrystalline **dppe** shows an isotropic line at –12.34 ppm, and at a spinning speed of 2 kHz, several sets of rotational sidebands that reflect the CSA (Chemical Shift Anisotropy) [23,24,51] of the signal. In the crystal, the surroundings of the phosphorus nuclei are well-defined, and the residual halfwidth of the isotropic line is only 330 Hz. In principle, there could be two isotropic lines; however, due to the arrangement of the molecules in the unit cell, the chemical shift difference does not lead to resolved signals [52,53].

The adsorption of **dppe** on the AC surface manifests in a substantial change of the ^31^P resonance (Figure 4, bottom). Due to the spiraling translational motion across the surface within the pores, the CSA is averaged out, and the rotational sidebands vanish. The linewidth of the signal increases to roughly 1.5 kHz because AC is an amorphous material, and the surroundings of the phosphorus nuclei is very heterogeneous. There is only one signal, which indicates that both phosphine groups in the **dppe** molecule are adsorbed at a surface coverage of 40%. Furthermore, there is no residual crystalline material inside or outside of the pores that would be obvious in the spectrum [37].

**Figure 5:**
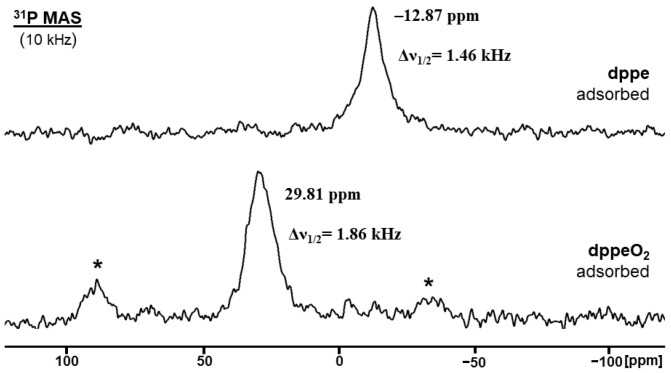
^31^P MAS NMR spectra of **dppe** adsorbed on AC with 40% surface coverage before (top) and after oxidation to **dppeO_2_** (bottom). The asterisks denote rotational sidebands of the bisphosphine dioxide signal.

The measurement of adsorbed **dppe** was performed under the exclusion of air (Figure 4). NMR measurements after admitting air into the sample flask show the immediate oxidation of the adsorbed bisphosphine. This indicates the importance of adsorption and mobility of the bisphosphines on the AC surface because polycrystalline **dppe** is stable in the atmosphere even after years of storage in the lab. After adsorption on AC with 40% surface coverage, the dioxide **dppeO_2_** forms within days. The chemical shift of the ^31^P MAS signal changes to 29.8 ppm, and the residual linewidth increases to 1.9 kHz (Figure 5). Both phosphine groups of **dppe** are oxidized, and no phosphine signal remains. Rotational sidebands reappear because phosphine oxides have a much larger CSA than phosphines [37,51]. The CSA of phosphine oxides is reduced upon adsorption but not entirely averaged out by the translational mobility and reorientation within the pores. This scenario has been observed earlier for bisphosphine dioxides with long alkyl spacers adsorbed on silica [37] and OPPh_3_ on silica and alumina. The obtained surface-adsorbed **dppeO_2_** is not oxidized any further to phosphinic or phosphonic acid species [54] upon prolonged exposure to the atmosphere.

**Figure 6:**
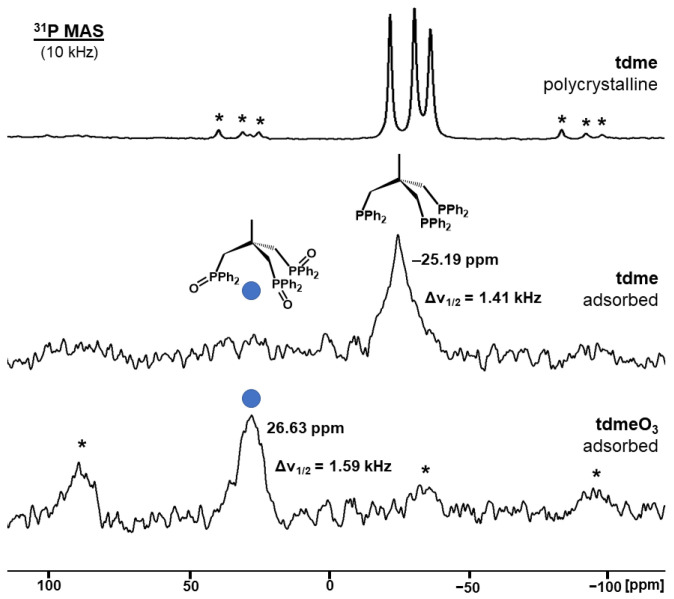
^31^P MAS NMR spectra of polycrystalline **tdme** (top), **tdme** adsorbed on AC with 25% surface coverage before oxidation (middle) and after oxidation to **tdmeO_3_** (bottom). The asterisks denote rotational sidebands.

In order to probe and broaden the range of phosphines amenable to the adsorption/oxidation process, we employed the trisphosphine **tdme** (Figure 6). Polycrystalline **tdme** results in three isotropic lines at −21.81 ppm, −30.54 ppm, and −36.27 ppm in the ^31^P MAS spectrum with the corresponding residual halfwidths of 217, 259, and 284 Hz. Each signal features one set of first order rotational sidebands (Figure 6, top). The presence of three isotropic lines with small halfwidths and very different chemical shifts has been observed previously for polycrystalline tripodal phosphines incorporating ethoxysilyl groups [55]. As in the latter case [55], this scenario is the result of three magnetically inequivalent phosphorus nuclei in the unit cell of **tdme** [56].

When **tdme** is applied to the AC surface with 40% surface coverage, all three phosphine groups are adsorbed and the ^31^P MAS spectrum only shows one signal (Figure 6, middle). The chemical shift of the signal at −25.19 ppm corresponds to the value obtained for **tdme** in solution. The CSA is averaged out for all phosphine signals of the trisphosphine. This is expected because the whole molecule is translationally mobile and not only the individual phosphine groups. As in the case of **dppe**, the residual linewidth of the **tdme** signal increases to 1.4 kHz upon adsorption because the phosphorus nuclei experience different surroundings on the amorphous AC surface.

Once the **tdme** adsorbed on AC is exposed to the atmosphere, the oxidation progresses, and eventually all phosphine groups are completely oxidized. The formation of **tdmeO_3_** is visible in the downfield shift of the ^31^P MAS signal to 26.63 ppm (Figure 6, bottom). The halfwidth grows to about 1.6 kHz. As in the case of **dppeO_2_**, the CSA increases again, and rotational sidebands come into existence in spite of the mobility of surface-adsorbed **tdmeO_3_** [37]. The complete oxidation of all phosphine groups is another indication of the mobility and contact of all the phosphorus atoms of **tdme** with the surface. It should be emphasized that **tdme** is air-stable and stored on the shelf in the polycrystalline form.

In summary, the solid-state ^31^P MAS spectra show that the bisphosphine **dppe** and the trisphosphine **tdme** are adsorbed and mobile on the AC surface and that they are quantitatively and selectively oxidized to **dppeO_2_** and **tdmeO_3_** after exposure to air. Side products can be excluded by comparison with ^31^P MAS spectra of adsorbed phosphine oxides [37], phosphinic and phosphonic acids [54], and polycrystalline phosphonium salts [57].

Adsorption on AC is an indispensable prerequisite for the surface-assisted oxidation of the bis- and trisphosphines. Therefore, the mobilities of the adsorbed phosphines have additionally been determined by their ^31^P T_1_ relaxation times. The polycrystalline and adsorbed **dppe** and **tdme** have been studied as representatives for bis- and trisphosphines. In general, the T_1_ times of polycrystalline molecules can be extremely long when CP (cross-polarization) [23,24] is not applied. For example, the ^31^P T_1_ relaxation time of polycrystalline PPh_3_ amounts to about 1000 s [58]. Due to the mobility of adsorbed species, the T_1_ times become orders of magnitude smaller. For example, PPh_3_, adsorbed on AC, has a ^31^P T_1_ time of only 0.16 s [22]. This value is even smaller than that obtained for PPh_3_ adsorbed on silica (0.59 s) [4]. Most probably, the paramagnetic nature of the AC is responsible for this difference [22].

Surface-adsorbed **dppe** with a coverage of 40% on AC exhibits a short T_1_ time of 0.15 s, as determined with an inversion recovery technique (Figures S1 and S2, Tables S2 and S3). This fast T_1_ relaxation additionally indicates the high translational mobility of **dppe** on the curved surface within the pores. The fact that only one specific T_1_ value was found also means that both phosphine groups show the same mobility. Therefore, a scenario where one phosphine group of the **dppe** molecule would be anchored on a specific surface site while the other would swirl around can be excluded.

The T_1_ relaxation time for **tdme** adsorbed on the AC surface with a coverage of 25% has been determined as 0.43 s (Figures S3 and S4, Tables S4 and S5). The lower surface coverage has been chosen to make sure that all phosphine groups can attach to the surface, considering that micropores of the AC might not be fully accessible to this larger molecule. The higher T_1_ value for **tdme**, as compared to the value of **dppe**, might reflect the presence of three phosphine groups that interact with the AC surface and slow down the motion of the tripodal phosphine. As in the case of **dppe**, only one T_1_ time was found for **tdme,** which means that the molecule moves as one unit across the AC surface.

In summary, the diminished CSA, the increased residual linewidths, and the significantly smaller ^31^P T_1_ values as compared to the polycrystalline materials indicate that **dppe** and **tdme** are mobile once they are adsorbed on the AC surface. As detailed below, this mobility correlates with the speed of oxidation of the adsorbed bis- and trisphosphines.

### 2.2. Monitoring the Oxidation of Phosphines on the AC Surface

#### 2.2.1. Methodology for Monitoring the Reaction

**Figure 7:**
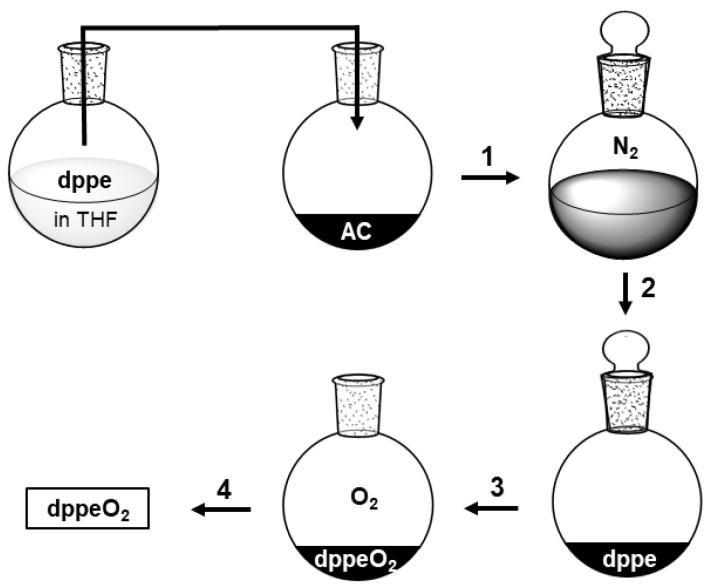
Adsorption and oxidation procedure of phosphines, outlined for dppe. **Step 1:** A solution of **dppe** in THF is combined with AC under a nitrogen atmosphere. **Step 2:** THF is removed in vacuo, and **dppe** adsorbs on the AC surface in a submonolayer. **Step 3:** Air is admitted, and the surface-adsorbed **dppe** is oxidized. **Step 4:** The product **dppeO_2_** is washed off of the AC surface with ethanol.

All phosphines used in this study self-adsorb when they are combined with AC and ground with a mortar and pestle. However, creating a mono- or submonolayer on the surface is faster when the phosphines are first dissolved, for example, in THF, and combined with the AC. The Subsequent removal of the solvent in vacuo results in the adsorbed phosphines (Figure 7). Care must be taken that no oxygen is admitted until the AC with the adsorbed phosphine is completely dry. Once air is admitted and the phosphine oxides are generated, they can be retrieved nearly quantitatively by washing them off of the AC surface.

As shown above, the adsorption and complete oxidation of the phosphines on the AC surface can be studied by classic solid-state NMR spectroscopy. However, the residual lines are rather broad due to the paramagnetism and amorphous nature of the AC. Additionally, the large CSA of phosphine oxides leads to second- and higher-order rotational sidebands. These are factors that ultimately reduce the spectral resolution and increase the measurement times. Therefore, for monitoring the progress of the oxidations, a faster spectroscopic method with much higher signal resolution is needed. In order to check the progress of the reaction frequently, a small sample of the AC with adsorbed phosphine and phosphine oxide can be taken and placed under an inert atmosphere. After adding THF, transferring the slurry into an NMR tube, and allowing the AC to settle, a ^31^P NMR spectrum reveals the presence and ratio of phosphine to phosphine oxide. For convenience and to avoid the formation of side products, the AC does not have to be filtered off prior to the ^31^P NMR measurements. In contrast to solid-state NMR, the attainable spectral resolution allows one to easily distinguish signals with similar chemical shifts and observe *J* coupling constants down to the 10 Hz range.

#### 2.2.2. Oxidation of Bis- and Trisphosphines Adsorbed on AC

Intrigued by the observation that PPh_3_ that is not air-sensitive [46] can be oxidized selectively in air once it is adsorbed on AC [22], we sought to expand the range of amenable phosphines to diverse bis- and trisphosphines (Figure 3). We chose **dppm** as an interesting candidate because the distance between the phosphine groups is small. This should increase the chance of detecting the simultaneous oxidation of both phosphines in one molecule. In other words, splitting one oxygen molecule should be favored in the case that the mechanism allows for this. The bisphosphine **dppm** is also the most rigid among the alkyldiarylphosphines chosen for this study.

The bisphosphines **dppe** and **dppp** allow one to identify any trend in reactivity and changes in the oxidation mechanism in case the distance between the phosphine groups and the flexibility of the backbone methylene bridges play important roles. The rigidity of the link between the phosphine groups is maximized with the phenyl ring in **dppbz**. With the latter bisphosphine, we intended to confirm that even the oxidation-resistant triarylphosphines undergo air oxidation once adsorbed on the AC surface. Furthermore, with **dppbz** a brush-type arrangement on the AC surface with only one of the phosphine groups touching the surface could take place. Similar stacking has been studied earlier using phosphonium formation on silica surfaces [59]. This stacking scenario of the rigid **dppbz** on AC could lead to the selective formation of the monoxide in cases of high surface coverages. Finally, the tripodal phosphine **tdme** was studied to check whether the first two phosphine groups within the molecule would be oxidized preferentially over the third one. A simultaneous oxidation mechanism (Figure 1, right) would favor the initial formation of the dioxide **tdmeO_2_**. In contrast to this, the consecutive mechanism would lead to three independent oxidation steps per molecule.

**Figure 8:**
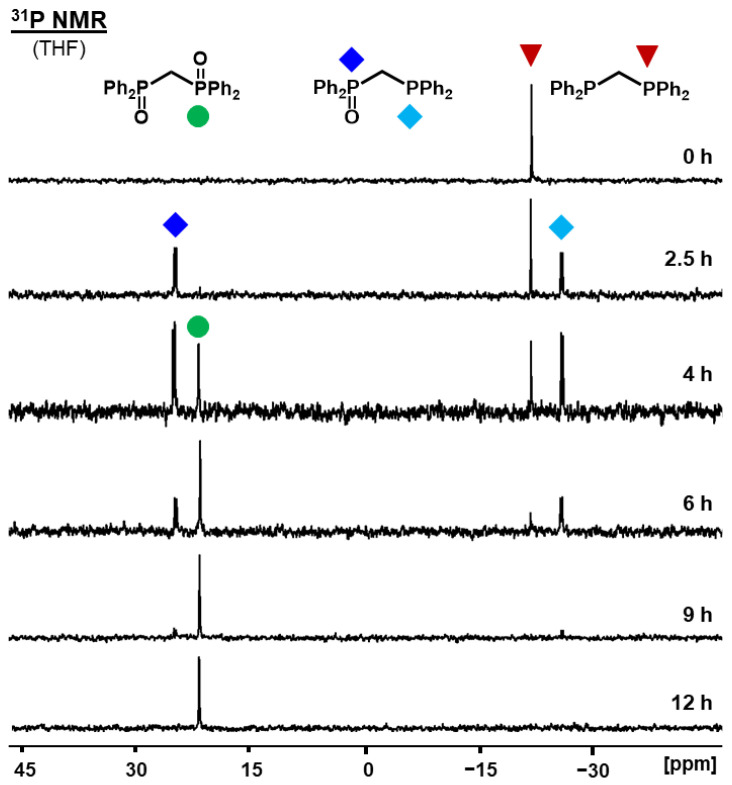
^31^P NMR spectra of adsorbed **dppm** on AC (40% surface coverage) measured after exposure to the atmosphere at the indicated times. The chemical shifts are 21.74 ppm for **dppmO_2_**, 25.00 and −26.16 ppm for **dppmO** (^2^*J*(^31^P-^31^P) = 51.5 Hz), and −22.08 ppm for **dppm**.

**Figure 9:**
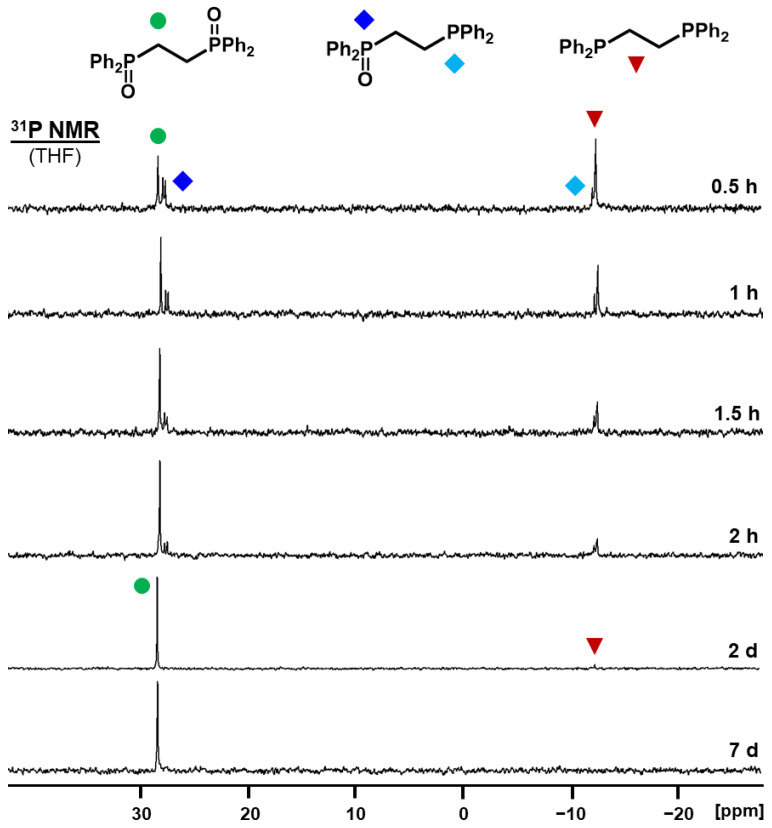
^31^P NMR spectra of adsorbed **dppe** on AC (40% surface coverage) measured after exposure to the atmosphere at the indicated times. The chemical shifts are 28.18 ppm for **dppeO_2_**, 27.62 and −12.20 ppm for **dppeO** (^3^*J*(^31^P-^31^P) = 48.1 Hz), and −12.41 ppm for **dppe**.

**Figure 10:**
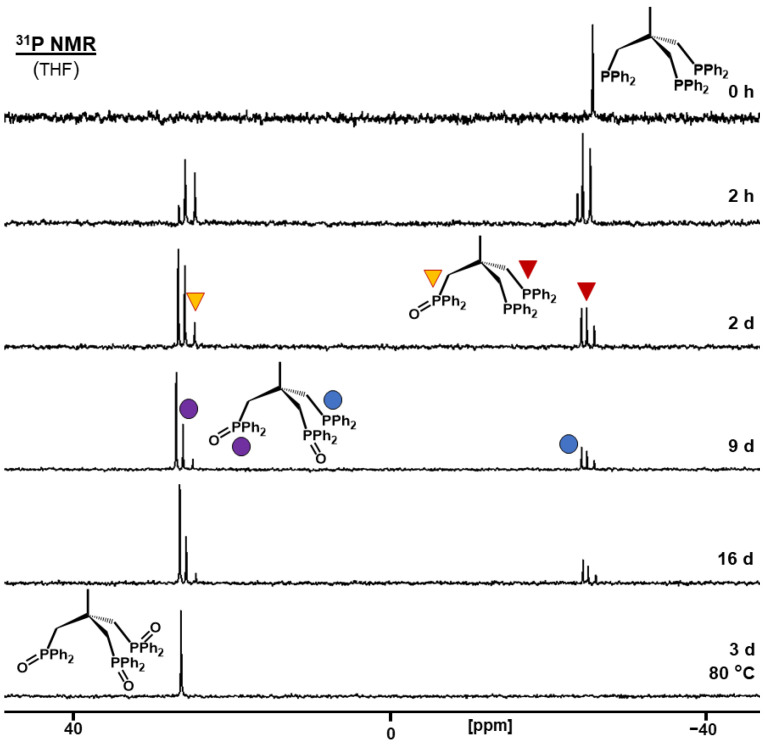
^31^P NMR spectra of adsorbed **tdme** on AC (40% surface coverage) measured after exposure to the atmosphere at the indicated times. The bottom spectrum was obtained after an additional 3 days of heating the sample to 80 °C. The chemical shifts are 26.66 ppm for **tdmeO_3_**, 25.88 and −24.11 ppm for **tdmeO_2_**, 24.63 and −24.77 ppm for **tdmeO**, and −25.74 ppm for **tdme**.

All phosphines are completely oxidized within 60 minutes when they are diluted on the surface with a low coverage of 10%. The corresponding ^31^P NMR spectra are shown in Figure S5. In order to study the oxidation process in detail and allow for all intermediates to be observed, a larger surface coverage of 40% was used to slow down the reaction. The ^31^P NMR spectra visualizing the oxidation progress for all bis- and trisphosphines are displayed in Figure 8, Figure 9 and Figure 10 and Figures S6–S10. The corresponding data are summarized in Tables S6–S9.

Regarding the spectral sequence in Figure 8 and the data summarized in Table S6, it is clear that the oxidation of each **dppm** molecule proceeds in a consecutive manner. The O_2_ molecule adsorbed on the AC surface is not split and taken up by one **dppm** molecule at once. In case **dppm** were transformed into **dppmO_2_** directly, the additional signals of **dppmO** would be absent in the spectrum series. The ^2^*J*(^31^P-^31^P) coupling of 52.4 Hz in the unsymmetric intermediate **dppmO** serves as an additional indicator that one phosphine oxide group per molecule is oxidized first. Consequently, the oxygen atoms of one O_2_ molecule must end up in different **dppm** molecules.

The observation about the oxidation of **dppm** is general in nature, as the same scenario is found for **dppe** and **dppp**. The spectra for the latter two are displayed in Figure 9 and Figure S6 and the conversion data are summarized in Tables S7 and S8. For **dppe,** the ^3^*J*(^31^P-^31^P) coupling of 48.1 Hz is again indicative of the initial generation of the unsymmetric monoxide **dppeO**. Compared to **dppm** and **dppe**, the oxidation of **dppp** is faster for the same surface coverage of 40% and complete within seven hours. Presumably, the longer, more flexible spacer with three methylene segments between the phosphine groups allows for higher mobility of the molecule on the AC surface [37].

While the bisphosphines clearly showed a stepwise oxidation of the two phosphines in the molecule, three intramolecular phosphine groups, as realized in **tdme**, could present a different scenario. The strong adsorption of all phosphine groups on the AC surface and limited mobility of the tripodal phosphine has been demonstrated by the solid-state NMR spectra above. In principle, this could increase the chance for the simultaneous oxidation of at least two of the phosphines within one **tdme** molecule. As the spectrum sequence displayed in Figure 10 shows, the oxidation still proceeds in a stepwise manner. The intensity of the monoxide signal peaks first, and **tdmeO_2_** follows. The progress of the oxidation stops after nine days at 40% surface coverage. However, the complete generation of **tdmeO_3_** can be achieved by heating the sample to 80 °C for three days. The higher temperature is still below the melting point, but it increases the translational mobility of the tripod, and the residual phosphine groups eventually find active oxygen on the AC surface. A lower surface coverage of 25% leads to complete oxidation of **tdme** to **tdmeO_3_** within 24 h (Figure S7). It should be noted that the lower surface coverage does not render the oxidation a simultaneous process, as the monoxide and dioxide intermediates are still observed.

Next, we tested whether a rigid spacer between the phosphine groups, as realized in **dppbz**, would allow the oxidation of both phosphine groups. Due to the possibility of upright stacking on the surface at high coverages [59], we were also curious whether the monoxide **dppbzO** could be obtained selectively. Prior to the AC-assisted oxidation, it had to be tested whether **dppbz** is air-sensitive. In contrast to PPh_3_, tetraphosphines like E(*p*-C_6_H_4_PPh_2_)_4_ (E = C, Si, Sn) featuring rigid scaffolds are slightly air-sensitive [59]. Figure S8 shows that **dppbz** is not oxidized in air as a neat solid, even after exposure in solution for several hours.

A high surface coverage of 92% of **dppbz** adsorbed on AC leads to only partial oxidation of the adsorbed bisphosphine even after prolonged exposure to the atmosphere for 6 days (Figure S9). Most probably, the rigid spacer leads to a brush-type stacking of the molecules on the surface at high loadings, but oxidation to **dppbzO_2_** still occurs. Unfortunately, the presence of three species discourages any attempt at selective monoxidation. However, with a lower surface coverage of 40%, complete oxidation to **tppbzO_2_** is observed within about 10 days (Figure S10). This result is highly significant also because **dppbz** is not air-sensitive and would otherwise require oxidants like H_2_O_2_ to cleanly produce the dioxide. Lower surface coverage results in faster oxidation, as outlined for the other bisphosphines with methylene bridges above.

### 2.3. Factors Influencing Phosphine Oxidation

The obvious impact of the surface coverage on the speed of the phosphine oxidation has already been explored previously [1] and discussed above. It has also been shown earlier that moisture on the AC surface has no influence on the oxidation rate of phosphines adsorbed on the surface [22]. Other important parameters that are investigated here include the effect of light, the pretreatment of the AC surface with an acid, the temperature, and the presence of radicals.

#### 2.3.1. Effect of Light on Phosphine Oxidation

Light can, in principle, impact or even enable the oxidation of phosphines adsorbed on AC surfaces. It has been described that light plays an important role in oxidation reactions in the presence of carbon-based networks [60,61]. For example, the use of light in combination with anthracene for alcohol oxidation has been reported [60]. The oxidation only proceeds with irradiation because excited states of reactants are needed for the process. Furthermore, the formation of phosphine radicals, followed by oxidation with molecular oxygen, depends on UV irradiation of 9,10-dicyanoanthracene [61].

**Figure 11:**
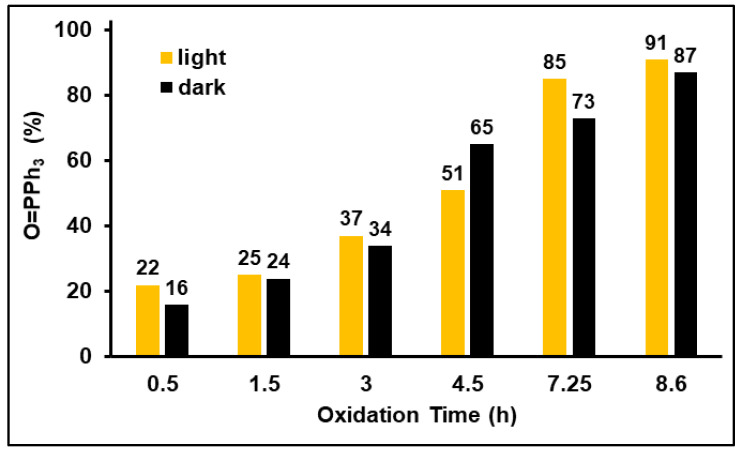
The oxidation rates of PPh_3_ adsorbed on AC exposed to daylight and in the dark show that light does not play any role in the oxidation.

Therefore, we tested the impact of light on the oxidation of phosphines adsorbed on AC. The results we obtained indicate that light has no effect on the oxidation of phosphines on the AC surface. As Figure 11 and Figure S11 show, the oxidation of PPh_3_ proceeds practically at the same rate as the control batch exposed to air in the dark.

#### 2.3.2. Effect of AC Acid Treatment on Phosphine Oxidation

AC is produced from different sources like wood, walnut or almond shells, and coal [7,8,9,62,63,64,65]. We reported earlier that AC contains phosphates due to the use of phosphoric acid in the production of AC [22,64]. While we could previously demonstrate that phosphates do not play a role in the AC-assisted oxidation of phosphines, they produce a ^31^P NMR background signal at 3 ppm [22]. In order to remove this resonance from the spectra of samples containing AC, the AC is vigorously washed with deionized water and thoroughly dried in vacuo before use [22]. Besides phosphates, other impurities in AC samples like traces of metals could be present that might catalyze the oxidation of phosphines on the surface [63,65]. While it has been shown earlier that most pure metal complexes do not catalyze the oxidation of phosphines [22], on the AC surface, this might be possible. Therefore, we washed the AC sample with an acid to remove any metal impurities on its surface.

**Figure 12:**
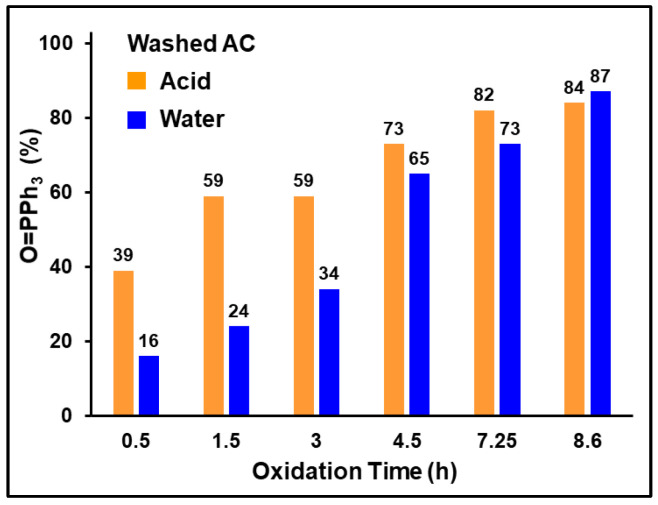
Oxidation of PPh_3_ adsorbed on AC with 40% surface coverage. One batch of the AC was washed with aqueous HCl, and the other one with water prior to use.

Stirring the AC with aqueous HCl for one day prior to the adsorption of PPh_3_ and exposure to air showed no significant change in the outcome of the oxidation (Figure 12 and Figure S12). Initially, acid washing of the AC enhanced the oxidation rate. It has been reported that acid treatment can not only remove impurities from the AC surface but also enhance the surface and pore accessibility [65]. This would explain the increase in phosphine oxidation at the start of the oxidation (Figure 12). If any metals were involved in the oxidation, removing them by the acid wash or at least decreasing their amount would have had a negative impact on the oxidation rate. Therefore, the observed outcome of this experiment rules out any metal-catalyzed oxidation. Furthermore, when PPh_3_ in solution is exposed to air in the presence of HCl, no oxidation occurs, rendering any acid involvement in the surface-assisted process less likely. It should also be noted that the unpaired electrons residing on the AC surface are not impacted by acid or water treatment. As the EPR spectra of AC batches washed with acid and water show, the resonance is slightly shifted, but its linewidth and shape are practically identical (Figure S13).

#### 2.3.3. Effect of Temperature on Phosphine Oxidation

While acid treatment of the AC and light exposure of the samples did not show any impact on the speed of oxidation of the adsorbed phosphines, the surface coverage and temperature are crucial. Both parameters have in common that they change the mobility of the adsorbed molecules on the surface. The higher the mobility, the faster the oxidation. A low surface coverage of 10% leads to a much faster oxidation of all phosphines within one hour (Figure S5), in contrast to 40% coverage, where the mobility of the adsorbed molecules is already impeded and more time is required.

Regarding the temperature, standardized conditions have been applied to study its impact on the surface-assisted air oxidation of phosphines. When **dppm** was adsorbed on AC with 40% surface coverage and exposed to air, the oxidation was complete within ten hours at ambient temperature (25 °C). Performing the same procedure at 80 °C led to complete oxidation of **dppm** within only four hours. It has to be noted that the melting point of **dppm** is 118–119 °C, and the observed surface adsorption and oxidation do not involve any melting process. When the AC sample with adsorbed **dppm** is cooled to 0 °C and exposed to air, the oxidation is completely stopped and does not progress, even after 24 h. The oxidation continues as soon as the sample is warmed up to room temperature. Being able to stop the oxidation on AC by simply placing the sample into a refrigerator allows the use of temperature as a convenient way to control the progress of the oxidation at any point. Most importantly, the impact of temperature on the oxidation indicates that the mobility of adsorbed **dppm** molecules on the AC surface plays a crucial role. We have demonstrated earlier for ferrocene adsorbed on AC that lower temperatures reduce the translational mobility [12]. With the **dppm** molecules being less mobile on the AC surface at lower temperatures, they cannot cruise and find adsorbed O_2_ molecules, and therefore, the oxidation does not proceed. This is one more indicator that O_2_ has to be adsorbed on the AC surface and activated prior to being able to oxidize the surface-adsorbed phosphines. It should be noted that in solution, air-sensitive phosphines are oxidized even at low temperatures.

#### 2.3.4. Oxidation of PPh_3_ in the Presence of AIBN

Next, we sought to investigate the role of radicals in the phosphine oxidation because the unpaired electrons on the AC surface are obviously important. EPR shows the presence of radicals on the AC surface (Figure S13) [22,66], and materials without unpaired electrons, such as silica or alumina, do not lead to phosphine oxidation [4]. Furthermore, the involvement of the unpaired electrons in the mechanism of the AC surface-assisted oxidation still needs to be explored further.

We added AIBN as a radical source to a solution of a phosphine to probe whether the oxidation with oxygen would be enabled by the unpaired electrons, even in the absence of the AC surface. The phosphine chosen for this test was PPh_3_, because, dissolved in THF, it cannot be oxidized in air even at elevated temperatures [22,46]. When AIBN is added to a THF solution of PPh_3_ in air, no oxidation is observed at room temperature. However, the complete oxidation of PPh_3_ to OPPh_3_ occurs when the solution is heated to 65 °C, the temperature at which AIBN creates radicals (Figure S14). In contrast, when a THF solution of AIBN and PPh_3_ was heated under the exclusion of O_2_, the AIBN radical formation alone did not lead to any OPPh_3_ or other P(V) species, even when heating the solution to 65 °C over prolonged times. This indicates that the O_2_ from air is responsible for the oxidation to OPPh_3_. Other P(V) species that could have been generated by the radicals and potential quaternization at the P atom can be excluded. This result is important because, incidentally, triarylalkylphosphonium salts exhibit signals at ^31^P NMR chemical shifts that are very similar to those of the corresponding phosphine oxides [57]. It should be noted that the presence of TEMPO as a radical source in a THF solution of PPh_3_ did not propagate the air oxidation to OPPh_3_. Naturally, TEMPO is a stable radical; however, the unpaired electrons on the AC surface are persistent as well, when the material is treated in different ways, for example, with acid (Figure S13).

From a synthesis point of view, the oxidation of phosphines on AC surfaces is superior to air oxidation using AIBN because AC is less expensive and the oxidation can be performed without a solvent and at room temperature. However, the fact that unpaired electrons indeed assist the selective air oxidation of phosphines supports the proposed sequence of steps in the oxidation process.

### 2.4. Phosphine Oxide Recovery

Regarding the selective oxidation of the bis- and trisphosphines to the corresponding monoxides, the surface-assisted synthesis offers some advantage. The oxidation can be stopped at an early stage, reducing the separation problem to removing the original phosphine from the monoxide. A statistical approach to oxidation with air or H_2_O_2_ would always lead to inseparable mixtures. However, the surface-assisted oxidation method still offers the advantage that the di- and trioxides are obtained nearly quantitatively without the need for reactive, potentially unsafe, and expensive oxidizers. Even the phosphines used in this study that are not air-sensitive, are oxidized selectively and quantitatively after adsorption on AC when exposed to the atmosphere. Most importantly, the phosphine oxides do not require any additional cleaning step for removing hydrogen-bonded H_2_O_2_ or water [43]. For example, the ^31^P, ^13^C, and ^1^H NMR spectra in Figure S15 show that **dppeO_2_** is obtained in its pure form. After simply washing the AC with THF or ethanol, **dppeO_2_** is obtained in an unoptimized yield of 88%.

### 2.5. Oxidation of (CO)_2_Ni(PPh_3_)_2_ on the AC Surface

**Figure 13:**
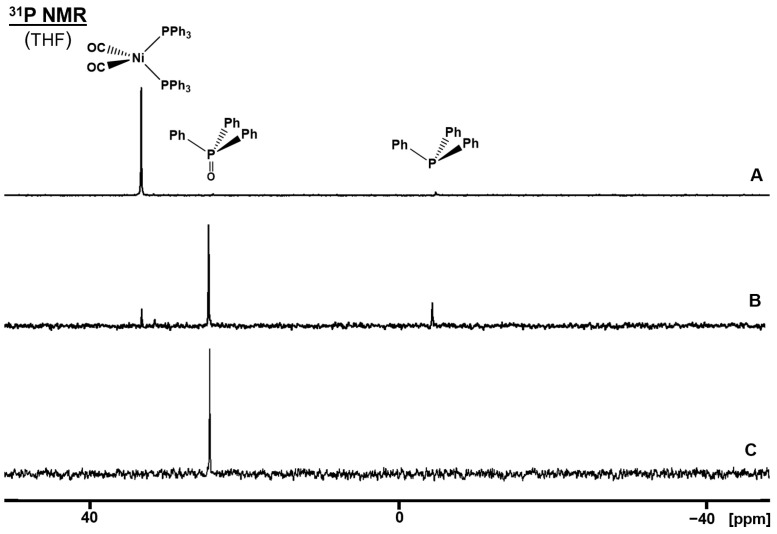
^31^ P NMR spectra of (CO)_2_Ni(PPh_3_)_2_ (**A**) in THF prior to oxidation, (**B**) adsorbed on AC with 40% surface coverage and exposed to air, and (**C**) the sample exposed to air for two days.

Finally, we sought to probe the implications of phosphine oxidation on AC surfaces in case the phosphines are coordinated to metal centers. This has immediate relevance for surface-supported and immobilized catalysts [67]. We described earlier that the robust, not air-sensitive complex (CO)_2_Ni(PPh_3_)_2_ loses the phosphine ligands on silica due to the driving force of surface adsorption [4]. However, no phosphine oxidation occurred on the silica surface. When (CO)_2_Ni(PPh_3_)_2_ is applied to the AC surface with 40% coverage and then the dry material is exposed to air, the PPh_3_ ligands are quantitatively oxidized to Ph_3_PO within two days (Figure 13, bottom). Prior to the completion of the oxidation, traces of uncoordinated PPh_3_ are visible in the ^31^P NMR spectrum (Figure 13, middle). This means that the surface adsorption is probably the driving force for ligand loss of the metal complex not only on silica [4] but also on the AC surface. This result shows that phosphine ligands coordinated to metal centers can potentially be removed from the complexes and self-adsorb on diverse surfaces. In the case of AC, complete oxidation of the phosphines takes place once air is admitted. On the one hand, this scenario must be considered when working with surface-supported or immobilized catalysts. On the other hand, the oxidation of the phosphine ligands will leave the Ni atoms on the surface and may potentially be utilized for single-atom catalyst preparation. Finally, this result demonstrates that even robust transition metal complexes containing phosphines should not be brought into contact with AC for purification purposes, as is common practice.

### 2.6. Mechanistic Considerations

**Figure 14:**
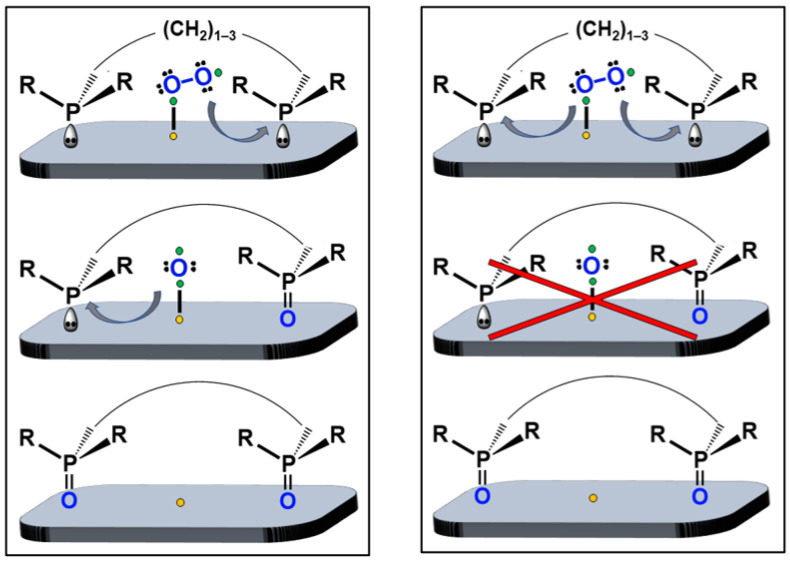
Consecutive (left) versus simultaneous (right) oxidation steps of a bisphosphine on the AC surface. Kinetic studies corroborate the consecutive sequence and render the simultaneous oxidation of both phosphine groups in the bisphosphine less likely because it does not feature a monoxide intermediate, as illustrated by the red cross.

As described in the Introduction section above, the previously reported surface-assisted oxidation of monophosphines described the indirect correlation of the surface coverage with the reaction rate [22]. Additionally, a steric influence and the need for surface-adsorbed O_2_ has been suggested [22]. Furthermore, delocalized unpaired electrons that reside in the aromatic ring systems on the surface of AC play a crucial role in the activation of the adsorbed O_2_ molecule [66,68]. Unfortunately, the AC surface is highly complex, and the way in which oxygen interacts with the surface is a matter of debate [69,70]. For example, the side-on versus end-on adsorption of the O_2_ molecule on graphitic materials has been investigated [69], as well as the need for defects on the surface [70]. The role of carbon sites at the edges of the aromatic systems and the formation of peroxo species on the AC surface have been studied as well [71]. Due to the complexity of the topic, we cannot claim that O_2_ is adsorbed in the end-on mode, as depicted in Figure 14. However, this detail is not relevant for the information we sought. Our focus was the key question of whether the O_2_ molecule is taken up in a concerted action by two phosphines in bis- and trisphosphines or in a stepwise manner, which may leave one oxygen atom as an intermediate on the AC surface. The latter could potentially be used for oxidizing other co-adsorbed species.

Unfortunately, using monophosphines, one cannot distinguish between the consecutive and simultaneous oxidation of phosphines with one O_2_ molecule (Figure 2). However, regarding the surface-assisted oxidation process of bisphosphines on the molecular level, two scenarios can be distinguished (Figure 14). The two phosphine groups of one bisphosphine could each take up one oxygen atom from O_2_ simultaneously. In this way, there would be no single oxygen atom left on the AC surface, not even as a transient species (Figure 14, right side, middle). Consequently, no phosphine monoxides should be detected as intermediates in this case. Alternatively, one oxygen atom can first be taken up by one phosphine group of the bisphosphine, and the surface-bound remaining oxygen atom could subsequently be scavenged by another phosphine group of the same or a different bisphosphine molecule (Figure 14, left). In this consecutive mechanism, phosphine monoxides should appear as intermediates. The presented results indicate that the oxidation of diverse bis- and trisphosphines follows the consecutive, stepwise process, because all monoxides could be observed easily. It should be noted that the dissolved monoxides are not oxidized further when exposed to air. This means that for oxidizing the second phosphine group within one molecule, the contact with the AC surface and activated O_2_ are indispensable.

**Figure 15:**
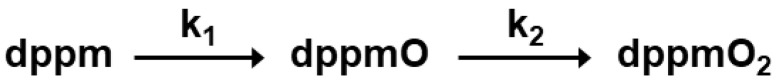
Oxidation steps of bisphosphine oxidation on the AC surface, depicted for **dppm** as an example.

**Figure 16:**
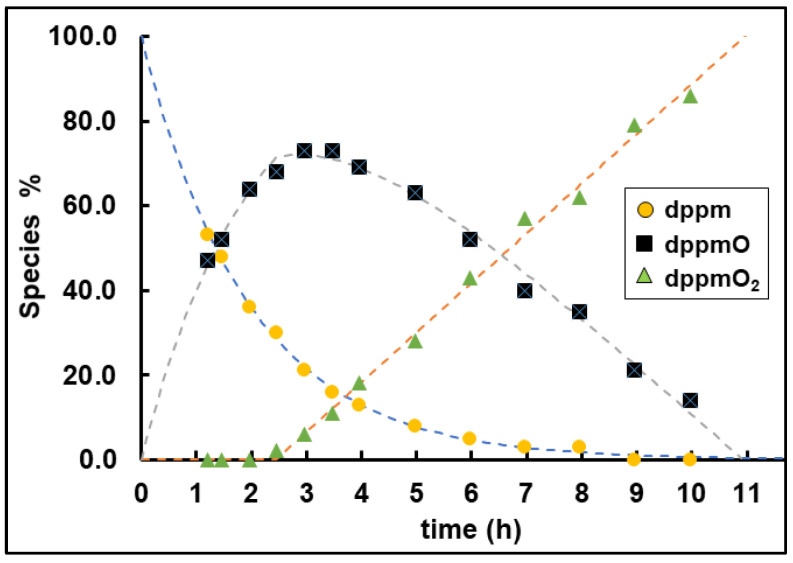
Graphical display of the data summarized in Table S6 for the oxidation of **dppm** after adsorption on AC with 40% surface coverage and exposure to air.

Our experiments indicate that oxygen has to be adsorbed on the AC surface first, and subsequently adsorbed phosphines are oxidized. Otherwise there would be no inverse correlation between the rate of oxidation and the surface coverage with adsorbed phosphines. One highly relevant scenario has been described recently for the oxidation of tertiary phosphines involving intermediate peroxy radicals [72]. In solution, P-O-O moieties form, which lead to the oxidation of another phosphine or to phosphinic acid esters [72]. The air oxidation of PPh_3_ in the presence of AIBN-derived radicals described above most probably follows this pathway. On the AC surface, P-O-O intermediates seem less likely when adsorbed phosphines are oxidized. No phosphinic acid esters have been detected in any of the experiments described in this contribution. Additionally, due to the proximity of the phosphine groups in the bisphosphines, the dioxides should be generated immediately. For example, in **dppm,** the intermediate Ph_2_PCH_2_P(OO)Ph_2_ would probably form the dioxide Ph_2_P(O)CH_2_P(O)Ph_2_ (**dppmO_2_**) instantaneously. Our results show the initial and exclusive formation of the monoxide **dppmO** (Figure 8 and Figure 15, and Figure 16), rendering the formation of peroxy radicals less likely. However, it could be argued that the orientation of the two phosphine groups within the adsorbed **dppm** molecule could impede the second step of any intramolecular oxidation and prolong the lifetime of a P-O-O intermediate until a second **dppm** molecule takes up one of its O atoms. Therefore, to clarify the existence of peroxy radicals on the AC surface, more studies will be needed in the future.

Using **dppm** as the example, the kinetics of the oxidation reaction could be studied in more detail (Figure 15). For this purpose, the signals of all species in the ^31^P NMR spectra (Figure 8) were integrated, and the conversion rates were calculated. The graphical displays obtained are shown for the oxidation of **dppm** (Figure 16). The oxidation of **dppm** proceeds in a stepwise manner with two different rate constants, k_1_ and k_2_ (Figure 15). First, the formation of **dppmO** occurs at a rate of 0.51 s^−1^ and after an induction period of 2.44 h **dppmO_2_** starts to appear (k_2_ = 11.8 M·s^−1^) (Figure 16).

Curve fitting of the decrease of **dppm** indicates that the first step of the reaction is first- or pseudo-first-order (Figure 16). Pseudo-first-order is expected since the concentration of adsorbed O_2_ on the AC surface is constant and the used-up oxygen will be replaced immediately by O_2_ uptake from the atmosphere. Furthermore, the footprint of the bisphosphine dioxide should be roughly the same as that of the bisphosphine, and therefore, the surface coverage should remain the same. Interestingly, the second step does not show a first-order but zeroth-order conversion of **dppmO** to **dppmO_2_**. The presence of an induction period in the formation of **dppmO_2_** could be an opportunity to selectively oxidize the bisphosphine to the monoxide and stop the reaction before any dioxide is formed. The monoxide could easily be separated from residual bisphosphine by column chromatography because phosphine oxides adsorb more strongly to silica and alumina surfaces than phosphines [4,37].

The data obtained for the oxidation of **dppp** (Figure S6, Table S8) show the same general trends with k_1_ = 0.44 s^−1^, k_2_ = 22.3 M·s^−1^, and an induction period of 0.86 h for the formation of **dpppO_2_**. The main difference compared to the oxidation of **dppm** is that the **dpppO_2_** forms more quickly. Consequently, the intermediate **dpppO** vanishes completely before **dpppO_2_** generation is complete. This makes the first step of the oxidation the limiting step in the overall rate. The end section of **dpppO_2_** formation tapers off as **dpppO** is all used up and now is dependent on the first step to move forward.

In summary, the kinetic data speak for the presence of two distinct steps in the oxidation process, as shown in Figure 15. For all bis- and trisphosphines, the monoxide forms first and then the dioxide and, in the case of **tdme**, the trioxide. Therefore, the consecutive, stepwise oxidation seems more likely (Figure 14, left). In future projects, it will be explored whether the oxygen atom residing on the AC surface as an intermediate can be employed for oxidizing other co-adsorbed species.

## 3. Conclusions

In the presented work, the bisphosphines **dppm**, **dppe**, and **dppp**, as well as one trisphosphine, **tdme**, and the triarylphosphines **dppbz** and PPh_3_, were adsorbed on AC surfaces and oxidized by air. The adsorption was studied by solid-state NMR spectroscopy, using the CSA, chemical shifts, residual halfwidths, and ^31^P T_1_ relaxation times as criteria. The progress of the oxidation was monitored by ^31^P solution and solid-state NMR spectroscopy. The effect of temperature on the oxidation process suggested that higher mobility of the phosphines on the surface increased the speed of oxidation. The oxidation is stopped at temperatures below 0 °C. More rigid phosphines with reduced mobility required longer oxidation times or lower surface coverages. It has been found that in our experiments, light had no effect on the speed of oxidation. Furthermore, the acid washing of the AC to remove metal impurities from the surface did not change the rate of phosphine oxidation. Kinetic studies using solution NMR analysis implied that the oxidation of multiple phosphine groups within one molecule proceeds in a consecutive manner. This result may be useful for producing selectively oxidized species in the future [73]. The oxidation of PPh_3_ using radicals created from AIBN in solution shows that unpaired electrons on the AC surface most probably play a crucial role in the oxidation of phosphines with air.

The oxidation of the phosphines in the metal complex (CO)_2_Ni(PPh_3_)_2_ shows that care should be taken when purifying solutions of metal complexes with AC, as is common practice. The removal of phosphines from metal centers has potential for creating single-atom catalysts on the surfaces of porous supports.

The presented research shows that clean phosphine oxides can be obtained using surface-assisted oxidation by simply exposing the adsorbed phosphines to the atmosphere. No expensive and hazardous oxidizers are required, and the phosphines can be retrieved from the AC surface in pure form. Since no aqueous hydrogen peroxide is involved, no water or H_2_O_2_ adducts form that would need to be destroyed in an additional purification step [43]. The stepwise oxidation of the bis- and trisphosphines suggests that the phosphine oxidation on AC creates activated oxygen atoms as intermediates directly on the surface or in the form of adsorbed P-O-O peroxy radicals [72] that may enable the surface-assisted co-oxidation of other substrates with the system AC/phosphines. In future projects, we will explore whether the latter enables other relevant oxidation reactions such as epoxidations or aldehyde oxidation.

In summary, the described research deepens the understanding of adsorption and AC surface-assisted air oxidation, and it offers a convenient method for the fast, easy, safe, and inexpensive direct synthesis of important clean phosphine oxides from diverse bis- and trisphosphines.

## 4. Experimental Section

**General Aspects and Activated Carbon.** The activated carbon (AC) DARCO (Fisher Scientific, specific surface area 650 m^2^/g) was used in this project. Other AC brands propagate the phosphine oxidation as well, but the time requirements differ somewhat, as described earlier [22]. Traces of phosphoric acid on the AC surface were removed by washing with water to avoid a background signal in the ^31^P NMR spectra. The AC was heated with copious amounts of deionized water to 60 °C, and the mixture was stirred for 3 h. After cooling, the slurry was filtered, and the AC was then placed in a Schlenk flask and dried at 100 °C under vacuum for about 3 h and stored under an inert gas atmosphere of purified nitrogen. The phosphines were applied as received. The amounts necessary for certain percentages of surface coverage were estimated as described previously [22]. A complete monolayer is declared as a 100% surface coverage in this contribution. The data for monolayer coverages of all phosphines, as well as (CO)_2_Ni(PPh_3_)_2_, are summarized in Table S1. Tetrahydrofuran (THF) was dried with a conventional solvent purification apparatus and kept over molecular sieves (4 Å). All reactions and handling of the materials were performed under a purified nitrogen atmosphere. The air that was admitted to the samples for oxidizing the adsorbed phosphines was not dried or otherwise purified.

**Solution NMR measurements.** About 30 mg of the AC containing adsorbed phosphine or phosphine oxide was scooped into a 5 mm NMR tube under a purified nitrogen atmosphere. By adding dry, oxygen-free THF, a suspension was created. After about 15 min the AC settled, and the samples were measured on a 500 MHz Varian NMR instrument. The ^31^P and ^13^C NMR spectra were recorded using routine pulse programs with ^1^H decoupling. The ^31^P measurements were performed using non-deuterated THF and disabling the lock function. As the ^31^P chemical shift standard, neat ClPPh_2_ in a capillary centered in the NMR tube was used (*δ*(^31^P) = +81.92 ppm). For ^1^H and ^13^C NMR, the solvent signals served as internal standards. Applying a line broadening factor of 10 Hz, ^31^P NMR spectra with a good S/N ratio were typically obtained with fewer than 256 scans.

**Solid-state NMR measurements.** For solid-state NMR measurements, the samples were packed into 4 mm ZrO_2_ rotors in air. All spectra were recorded on a Bruker Avance 400 solid-state NMR spectrometer. The ^31^P NMR spectra were recorded using standard single-pulse programs with high-power proton decoupling and a 2 s pulse delay. Fewer than 256 scans were required to achieve a sufficient S/N ratio when a line-broadening factor of 50 Hz was applied. The T_1_ times were determined with a Bruker inversion recovery pulse program at a spinning speed of 10 kHz to maximize the S/N ratio. The ^31^P MAS NMR spectra were calibrated with (NH_4_)_2_HPO_4_ (*δ*(^31^P) = +0.81 ppm) as the external standard.

**EPR measurements.** The EPR spectra were recorded at ambient temperature using the instrument Bruker Elexsys E500. The powdered samples were thoroughly dried and placed into the EPR tubes under the atmosphere. The magnetic field was centered at 3350 Gauss, and the sweep width spanned from 3200 to 3500 Gauss. With 1024 points and a sample g-factor of 2.000, 32 scans using a continual wave sweep were sufficient to result in good spectrum quality.

**Adsorption of phosphines on AC.** In a representative experiment, AC (1.80 g) was placed in a Schlenk flask. The bisphosphine **dppm** (260 mg, 0.68 mmol, 41% surface coverage) was dissolved in 10 mL of THF in a separate flask, and the solution was added to the AC. The slurry was stirred for 20 min to allow for the phosphine solution to enter the pores and the phosphine to adsorb on the AC surface in a submonolayer. The THF was then removed slowly under vacuum at RT until the AC was thoroughly dried within about 30 min. All steps were performed under the exclusion of oxygen using Schlenk techniques. The samples were exposed to the atmosphere only for the oxidations to take place.

**Monitoring the oxidation of adsorbed phosphines.** To check the oxidation progress of the adsorbed phosphines multiple times, the AC sample loaded with the phosphine was exposed to the atmosphere. This was accomplished by simply removing the stopper of the Schlenk flask (Figure 7). Exposing the loaded AC batch on a watch glass did not lead to faster oxidation. Over time, portions of the dry AC were filled into a 5 mm NMR tube. Dry, oxygen-free THF was added, and the sample was measured after the AC settled. Non-deuterated THF was used while the lock function of the spectrometer was disabled.

**Investigating the effect of light.** A batch of AC containing adsorbed PPh_3_ with 40% surface coverage was prepared. Twelve aliquots with about equal amounts of the AC were scooped into 12 NMR tubes. Six samples were placed under fluorescent and daylight and the other six NMR tubes were covered and kept in the dark during the oxidation. The progress of the oxidation under light and in the dark was monitored by adding THF to two sample tubes at the same time intervals, one from the series kept in the dark, and one exposed to the light during oxidation. Measurement by ^31^P NMR and the integration of the phosphine and phosphine oxide signals allowed us to compare the progress of oxidation in the light and dark during the same time intervals.

**Washing the AC with acid.** AC (500 mg) was stirred in aqueous HCl (10%, 50 mL) for 20 h at RT. Then the mixture was gravity filtered through filter paper. The AC was returned to the flask and stirred with deionized water (100 mL) at 50 °C for 3.5 h. The suspension was gravity-filtered, and the AC was returned to the flask and washed a second time with deionized water (100 mL, 60 °C, 16 h). The supernatant was tested with indicator paper, which showed a pH of about 5. After the AC was washed with water one more time (100 mL, 75 °C, 3 h), the pH of the filtrate was 7. The AC was rinsed with ethanol and heated to 75 °C for 2 h under vacuum until it was completely dry.

**Recovery of the phosphine oxides.** The recovery of the phosphine oxides is described using **dppe** adsorbed with 40% surface coverage on about 150 mg of AC as an example. After the complete oxidation of the surface-adsorbed **dppe** to **dppeO_2_** within 24 h, 10 mL of ethanol was added to the dry AC sample, and the mixture was stirred for 30 min. The suspension was then filtered, and the filtrate was collected. The AC was transferred back into the original flask and washed two more times with 10 mL aliquots of ethanol. After the completion of the washing cycles, the solvent was removed from the combined filtrates in vacuo to yield 87.5% of the phosphine oxide **dppeO_2_**. The purity of the product was confirmed with ^1^H, ^13^C, and ^31^P NMR spectroscopy (Figure S15).

## Data Availability

The original contributions presented in this study are included in the article/supplementary material. Further inquiries can be directed to the corresponding author.

## Supplementary Materials

The following supporting information can be downloaded at: https://www.mdpi.com/article/10.3390/molecules30132737/s1. Table S1: Amounts of phosphines used to create a monolayer on the AC surface. In this contribution a monolayer is referred to as 100% surface coverage; Figure S1: ^31^P MAS NMR spectra acquired with a 180°–τ–90° inversion recovery pulse program for **dppe** adsorbed on AC with 40% surface coverage; Table S2: Data for the inversion recovery NMR spectra of **dppe** (adsorbed on AC with 40% surface coverage) in Figure S1, used for the fitting in Figure S2. The delay times τ between the 180° and 90° pulses are matched with the intensities of the resulting spectra which were measured using TopSpin software with arbitrary units. The data was normalized with every intensity value divided by the intensity value for a delay time of 8 s; Figure S2: Experimental data for **dppe**, adsorbed on AC with 40% surface coverage, taken from Table S2 (black dots) and the fit (grey dotted line) calculated from the data using LabPlot software; Table S3: Parameters and error information for the fit generated from the experimental data in Table S2 used to calculate the ^31^P T_1_ relaxation time for **dppe** adsorbed on AC with 40% surface coverage; Figure S3: ^31^P MAS NMR spectra acquired with a 180°–τ–90° inversion recovery pulse program for **tdme** adsorbed on AC with 25% surface coverage; Table S4: Data for the inversion recovery NMR spectra of **tdme** (adsorbed on AC with 25% surface coverage) in Figure S3, used for the fitting in Figure S4. The delay times τ between the 180° and 90° pulses are matched with the intensities of the resulting spectra which were measured using TopSpin software with arbitrary units. The data was normalized with every intensity value divided by the intensity value for a delay time of 8 s; Figure S4: Experimental data for **tdme**, adsorbed on AC with 25% surface coverage, taken from Table S4 (black dots) and the fit (grey dotted line) calculated from the data using LabPlot software; Table S5: Parameters and error information for the fit generated from the experimental data in Table S4 used to calculate the ^31^P T_1_ relaxation time for **tdme** adsorbed on AC with 25% surface coverage; Figure S5: ^31^P NMR spectra of **dppe** and **dppp**, adsorbed on AC with 10% surface coverage. After exposure of the samples to the atmosphere for one hour, complete oxidation to **dppeO_2_** and **dpppO_2_** is found; Table S6: Presence of **dppm**, the monoxide **dppmO**, and **dppmO_2_** after the indicated times of air exposure of **dppm** adsorbed on AC with 40% surface coverage. The values are based on the integration of the ^31^P NMR signals; Table S7: Presence of **dppe**, the monoxide **dppeO**, and **dppeO_2_** after the indicated times of air exposure of **dppe** adsorbed on AC with 40% surface coverage. The values are based on the integration of the ^31^P NMR signals; Figure S6: ^31^P NMR spectra of adsorbed **dppp** on AC (40% surface coverage) measured after exposure to the atmosphere at the indicated times. The chemical shifts are 28.00 ppm for **dpppO_2_**, 27.14 and −17.40 ppm for **dpppO**, and −17.29 ppm for **dppp**; Table S8: Product yields based on the integration of the ^31^P NMR signals after exposure of the adsorbed **dppp** (40% surface coverage) to the atmosphere at the indicated times; Figure S7: ^31^P NMR spectra of **tdme** adsorbed on AC with 25% surface coverage and measured after exposure to the atmosphere at the indicated times. The chemical shifts are 26.66 ppm for **tdmeO_3_**, 25.88 and −24.11 ppm for **tdmeO_2_**, 24.63 and −24.77 ppm for **tdmeO** and −25.74 ppm for **tdme**; Figure S8: ^31^P NMR spectra of **dppbz** dissolved in THF and exposed to air for 0.5 and 2.5 h (top two spectra) and ^31^P MAS spectrum of neat, polycrystalline **dppbz** exposed to the atmosphere for 22 h (bottom); Figure S9: ^31^P NMR spectra of **dppbz** adsorbed on AC with 92% surface coverage and exposed to air for 1 (top) and 6 days (bottom). The chemical shifts are 23.82 ppm for **dppbzO_2_**, 24.06 and −4.64 ppm for **dppbzO**, and −5.34 ppm for **dppbz**; Figure S10: ^31^P NMR spectra of **dppbz** adsorbed on AC with 40% surface coverage and measured after exposure to the atmosphere at the indicated times. The chemical shifts are 23.82 ppm for **dppbzO_2_**, 24.06 and −4.64 ppm for **dppbzO**, and −5.34 ppm for **dppbz**; Table S9: Presence of **dppbz**, the monoxide **dppbzO**, and **dppbzO_2_** after the indicated times of air exposure of **dppbz** adsorbed on AC with 40% surface coverage (*92% surface coverage; exposure times one and six days). The values are based on the integration of the ^31^P NMR signals; Figure S11: ^31^P NMR spectra for monitoring the oxidation of PPh_3_ adsorbed with 40% surface coverage on AC in the dark (left) and exposed to daylight (right). Samples were taken from both batches at the indicated times; Figure S12: ^31^P NMR spectra for monitoring the oxidation of PPh_3_ adsorbed with 40% surface coverage on AC. The AC has been washed with water prior to use (left) and with aqueous HCl (right). Samples were taken from both batches at the indicated times; Figure S13: EPR spectra of AC after washing with HCl (red line) and with water (blue line); Figure S14: ^31^P NMR spectra of a solution of PPh_3_ in THF stirred at 65 °C for four hours under the atmosphere (**A**) and the same solution after adding 10 mol% AIBN and stirring for four hours under identical conditions (**B**); Figure S15: ^31^P (top), ^13^C (middle), and ^1^H (bottom) NMR spectra of **dppeO_2_** obtained quantitatively after adsorption on AC with 40% surface coverage and exposure to the atmosphere. All ^13^C NMR signals, except that of C*_p_* show virtual couplings.
