# Supplementary material for: Selective Air Oxidation of Bis- and Trisphosphines Adsorbed on Activated Carbon Surfaces
Source: Molecules. 2025 Jun 25;30(13):2737. doi: 10.3390/molecules30132737 (PMC12251510; doi:10.3390/molecules30132737)
Supplement: Supplementary file 1 [file molecules-30-02737-s001.zip › molecules-3696520-supplementary.pdf]

Supplementary Information (SI)

Selective Air Oxidation of Bis- and Trisphosphines Adsorbed on Activated Carbon Surfaces

Ehsan Shakeri, John C. Hoefler, Janet Blümel*

Department of Chemistry, Texas A&M University, College Station, TX, 77842-3012, USA.
Email: bluemel@tamu.edu

Table S1. Amounts of phosphines used to create a monolayer on the AC surface. In this contribution a monolayer is referred to as 100% surface coverage.

**Phosphine**	**100% Surface Coverage (mg/g AC)**	**100% Surface Coverage (mmol/g AC)**
**dppm**	350	0.92
**dppe**	290	0.72
**dppp**	270	0.64
**dppbz**	300	0.68
**tdme**	410	0.66
**PPh_3**	440	1.70
**$(CO)_2Ni(PPh_3)_2$**	490	0.77

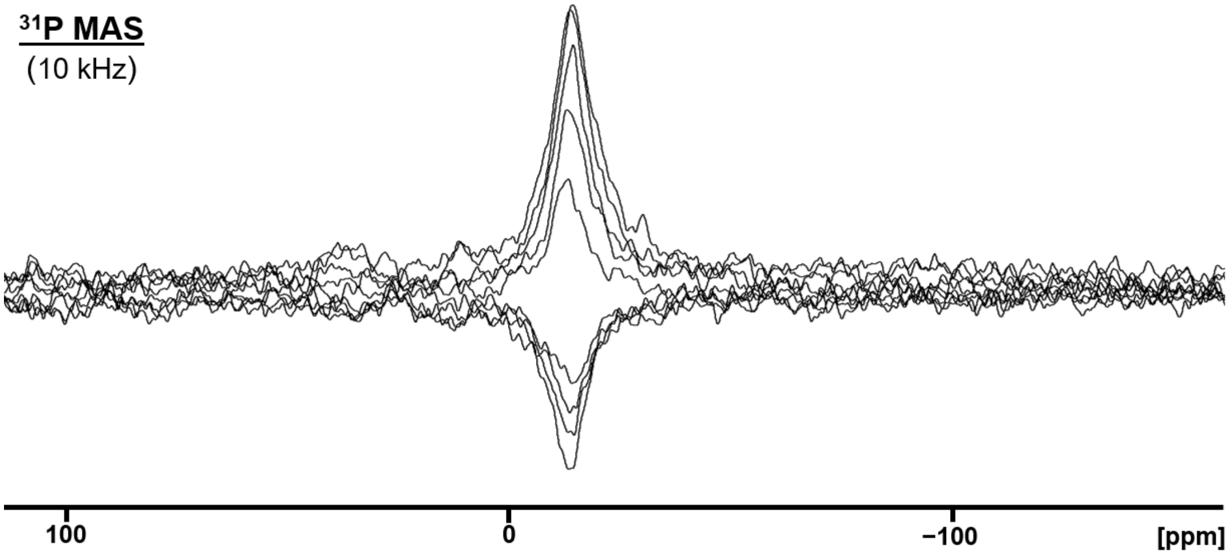

Figure S1. ^{31}P MAS NMR spectra acquired with a 180°–τ–90° inversion recovery pulse program for **dppe** adsorbed on AC with 40% surface coverage.

Table S2. Data for the inversion recovery NMR spectra of **dppe** (adsorbed on AC with 40% surface coverage) in Figure S1, used for the fitting in Figure S2. The delay times τ between the 180° and 90° pulses are matched with the intensities of the resulting spectra which were measured using TopSpin software with arbitrary units. The data was normalized with every intensity value divided by the intensity value for a delay time of 8 seconds.

**Delay Time τ (s)**	**Intensity**
0.001	–0.620
0.01	–0.489
0.03	–0.421
0.05	–0.313
0.3	0.392
0.5	0.627
1	0.839
5	0.983
8	1.00

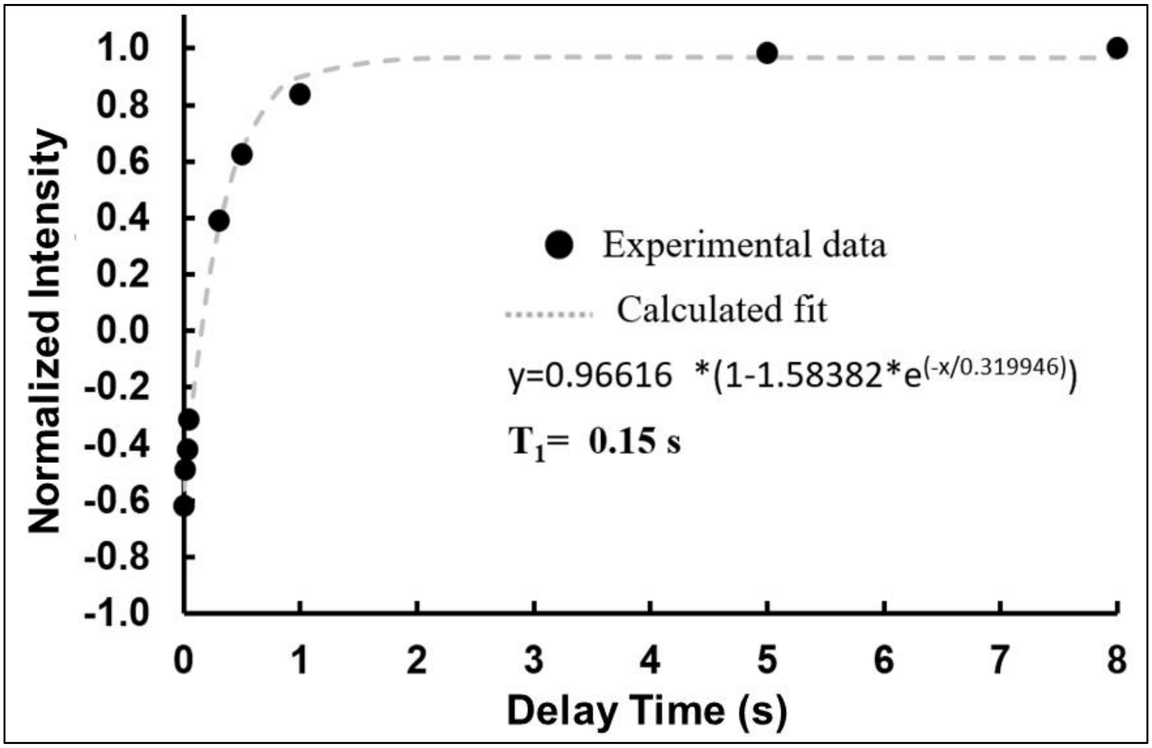

Figure S2. Experimental data for **dppe**, adsorbed on AC with 40% surface coverage, taken from Table S2 (black dots) and the fit (grey dotted line) calculated from the data using LabPlot software.

Table S3. Parameters and error information for the fit generated from the experimental data in Table S2 used to calculate the ^{31}P T_1 relaxation time for **dppe** adsorbed on AC with 40% surface coverage.

Equation: $y = \mathbf{A} \cdot (1 - \mathbf{B} \cdot e^{-x/T})$							
Variable	Value	Uncertainty	Uncertainty (%)	t statistic	P > |t|	Upper	Lower
A	0.96616	0.0276139	2.86	3.50E+01	3.63E-08	1.03373	0.898592
B	1.58382	0.0324889	2.05	48.7	5.00E-09	1.66332	1.50432
T	0.319946	0.0233127	7.29	1.37E+01	9.30E-06	0.37699	0.262902

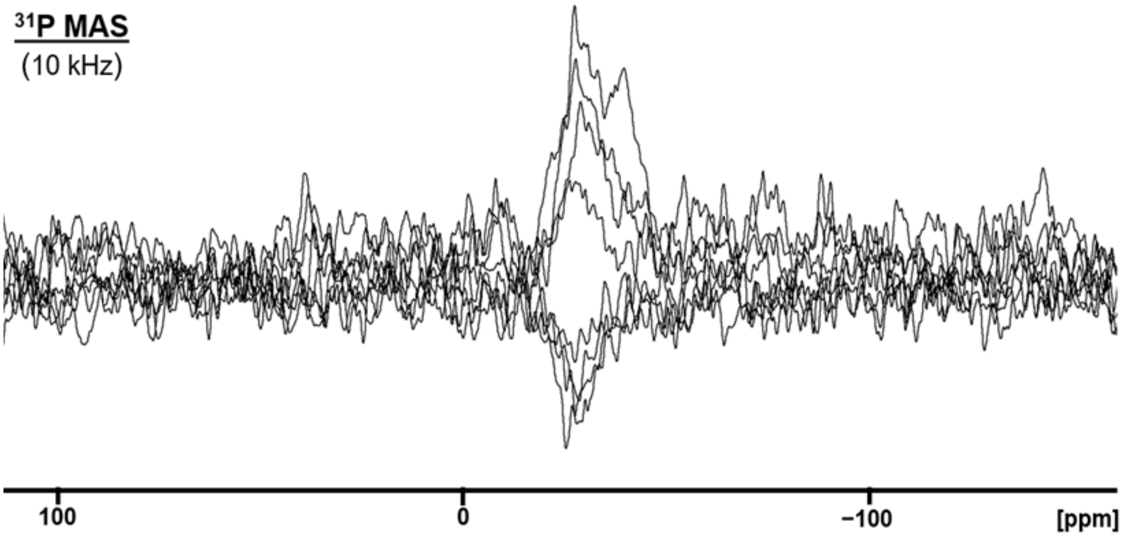

Figure S3. ^{31}P MAS NMR spectra acquired with a 180°–τ–90° inversion recovery pulse program for **tdme** adsorbed on AC with 25% surface coverage.

Table S4. Data for the inversion recovery NMR spectra of **tdme** (adsorbed on AC with 25% surface coverage) in Figure S3, used for the fitting in Figure S4. The delay times τ between the 180° and 90° pulses are matched with the intensities of the resulting spectra which were measured using TopSpin software with arbitrary units. The data was normalized with every intensity value divided by the intensity value for a delay time of 8 seconds.

**Delay Time τ (s)**	**Intensity**
0.001	–0.725
0.01	–0.655
0.05	–0.534
0.1	–0.372
1	0.460
2.5	0.789
4	0.877
8	1.00

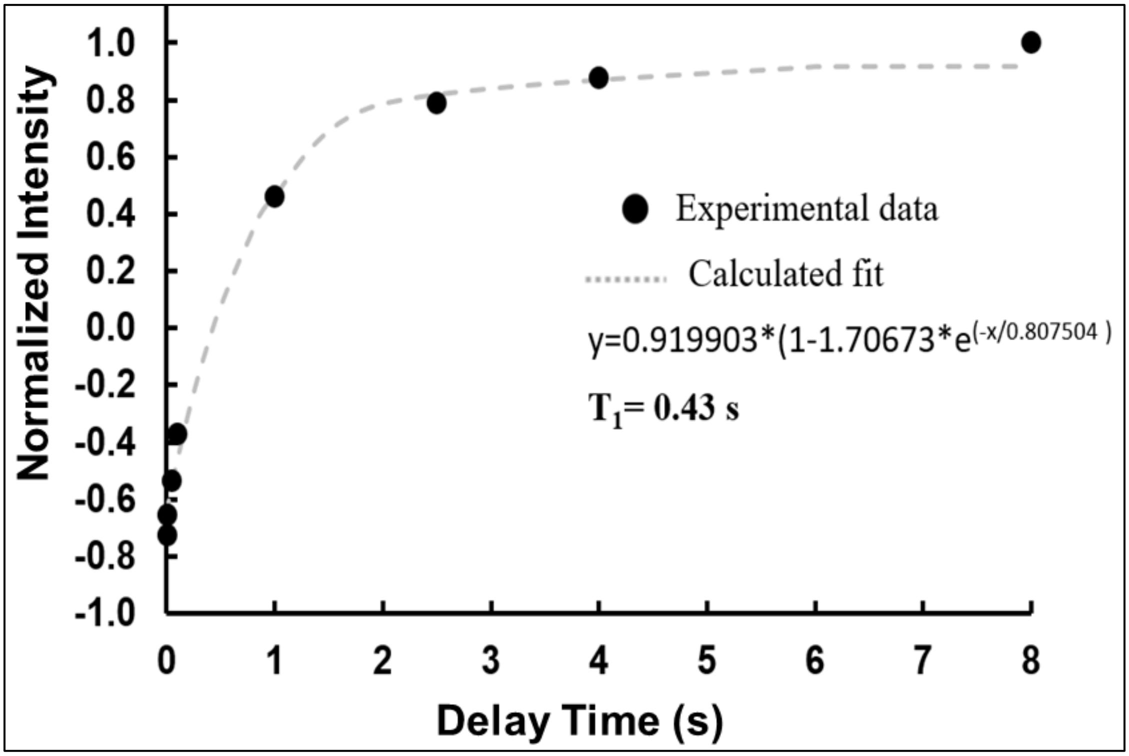

Figure S4. Experimental data for **tdme**, adsorbed on AC with 25% surface coverage, taken from Table S4 (black dots) and the fit (grey dotted line) calculated from the data using LabPlot software.

Table S5. Parameters and error information for the fit generated from the experimental data in Table S4 used to calculate the ^{31}P T_1 relaxation time for **tdme** adsorbed on AC with 25% surface coverage.

Equation: $y = \mathbf{A}\cdot(1-\mathbf{B}\cdot e^{-x/T})$							
Variable	Value	Uncertainty	Uncertainty (%)	t statistic	P > |t|	Upper	Lower
A	0.919903	0.0553603	6.02	16.6	7.68E-0.5	1.07361	0.766199
B	1.70673	0.0691282	4.05	24.7	1.6E-05	1.89866	1.5148
T	0.807504	0.137342	17	5.88	0.00418	1.18883	0.426182

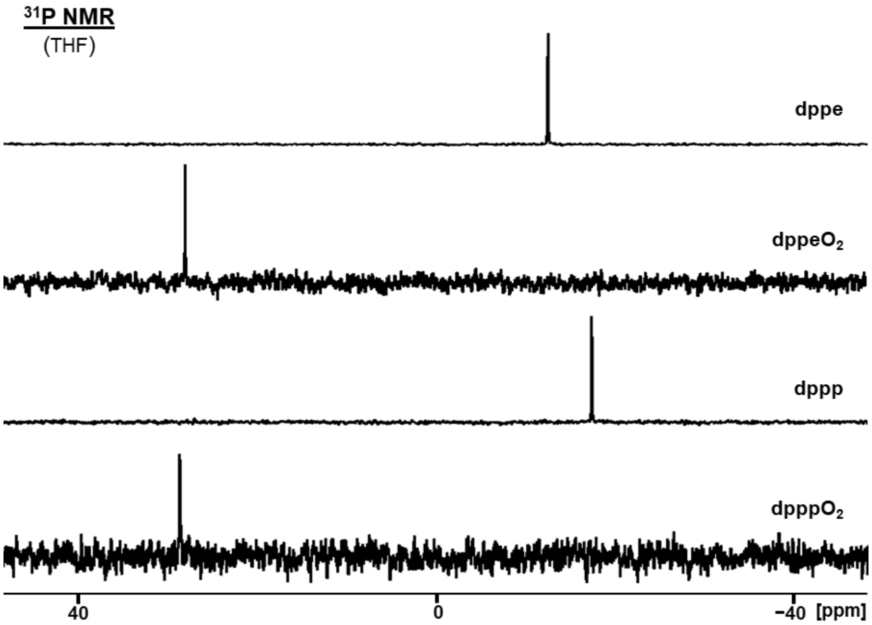

Figure S5. ^{31}P NMR spectra of **dppe** and **dppp**, adsorbed on AC with 10% surface coverage. After exposure of the samples to the atmosphere for one hour, complete oxidation to **dppeO$_2$** and **dpppO$_2$** is found.

Table S6. Presence of **dppm**, the monoxide **dppmO**, and **dppmO$_2$** after the indicated times of air exposure of **dppm** adsorbed on AC with 40% surface coverage. The values are based on the integration of the ^{31}P NMR signals.

Time (h)	dppm (%)	dppmO (%)	dppmO$_2$ (%)
0	100	0	0
1.2	53	47	0
1.5	48	52	0
2	36	64	0
2.5	30	68	2
3	21	73	6
3.5	16	73	11
4	13	69	18
5	8	64	28
6	5	52	43
7	3	40	57
8	3	35	62
9	0	21	79
10	0	14	86
12	0	0	100

Table S7. Presence of **dppe**, the monoxide **dppeO**, and **dppeO$_2$** after the indicated times of air exposure of **dppe** adsorbed on AC with 40% surface coverage. The values are based on the integration of the ^{31}P NMR signals.

Time	dppe (%)	dppeO (%)	dppeO$_2$ (%)
0.5 h	27	47	26
1 h	26	39	35
1.5 h	15	38	47
2 h	10	27	63
2 d	4	0	96
7 d	0	0	100

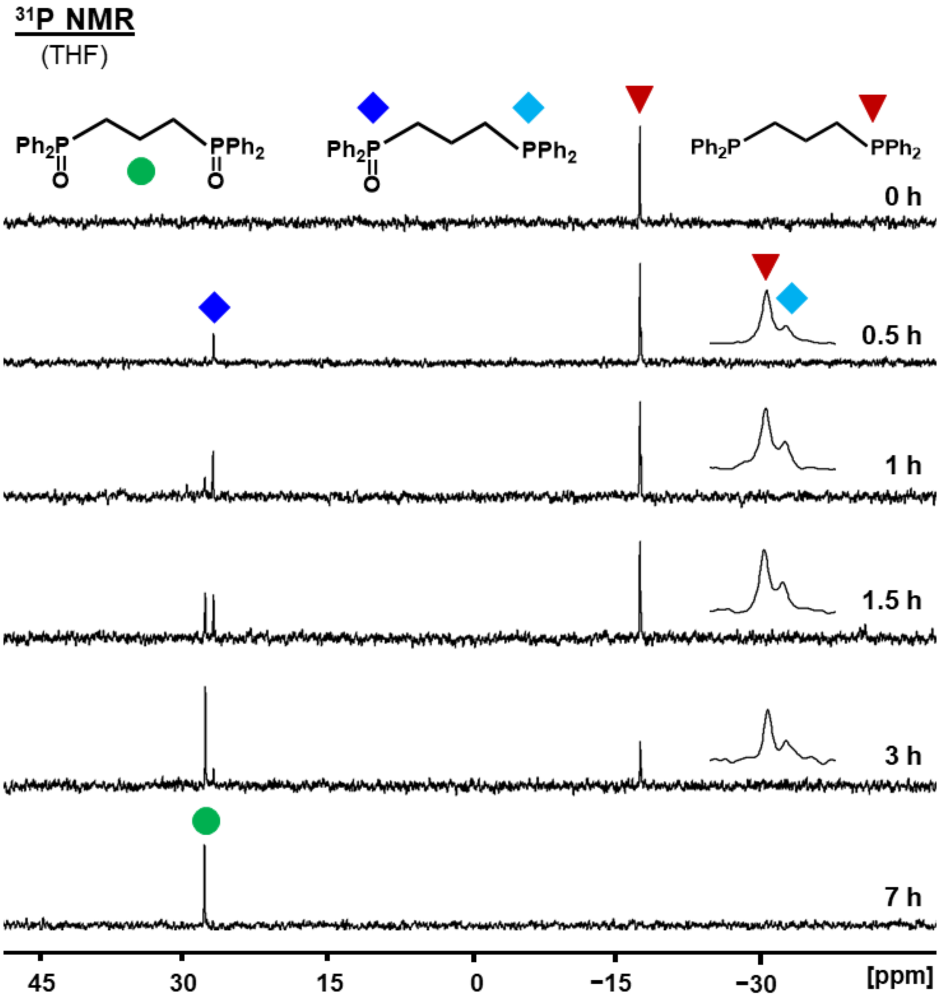

Figure S6. ^{31}P NMR spectra of adsorbed **dppp** on AC (40% surface coverage) measured after exposure to the atmosphere at the indicated times. The chemical shifts are 28.00 ppm for **dpppO$_2$**, 27.14 and –17.40 ppm for **dpppO**, and –17.29 ppm for **dppp**.

Table S8. Product yields based on the integration of the ^{31}P NMR signals after exposure of the adsorbed **dppp** (40% surface coverage) to the atmosphere at the indicated times.

Time (h)	dppp (%)	dpppO (%)	$dpppO_2$ (%)
0	100	0	0
0.25	78	22	0
0.5	63	37	0
1	43	44	13
1.5	41	33	26
2	34	29	37
3	26	16	58
4	15	0	85
5	9	0	91
7	0	0	100

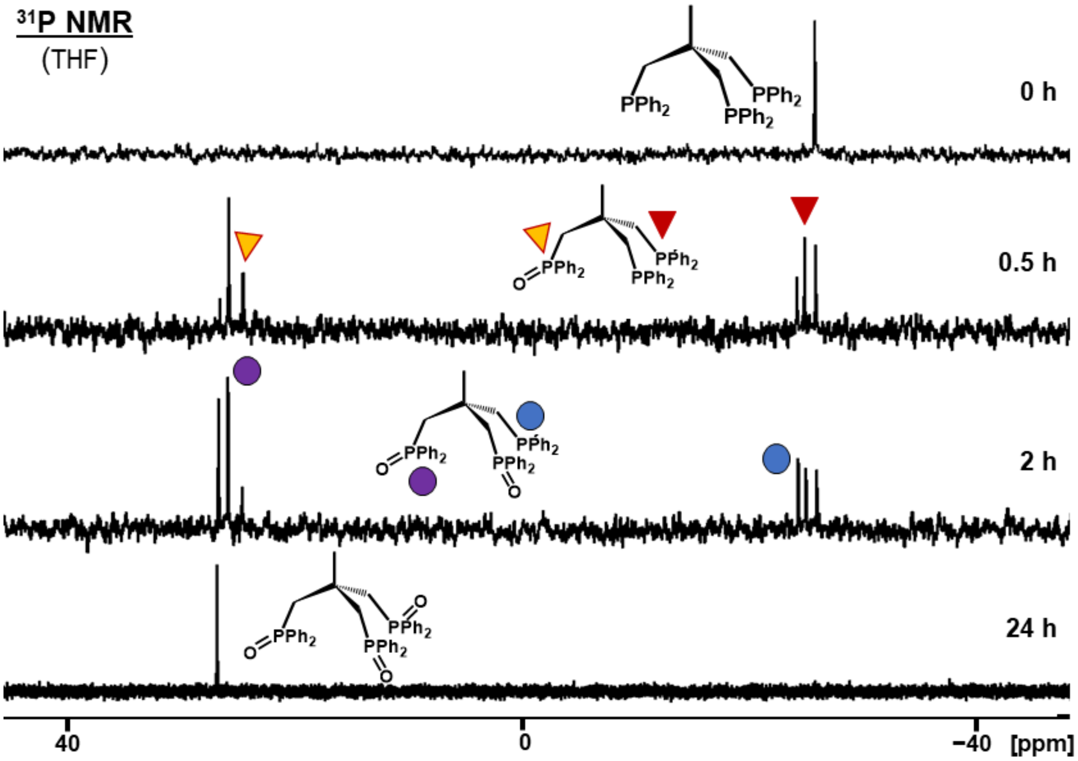

Figure S7. ^{31}P NMR spectra of **tdme** adsorbed on AC with 25% surface coverage and measured after exposure to the atmosphere at the indicated times. The chemical shifts are 26.66 ppm for **$tdmeO_3$**, 25.88 and –24.11 ppm for **$tdmeO_2$**, 24.63 and –24.77 ppm for **tdmeO** and –25.74 ppm for **tdme**.

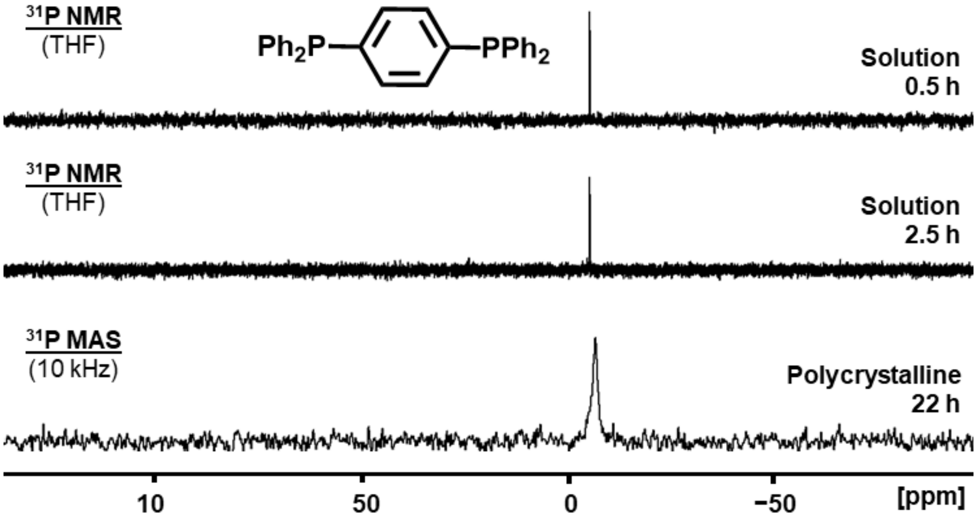

Figure S8. ^{31}P NMR spectra of **dppbz** dissolved in THF and exposed to air for 0.5 and 2.5 hours (top two spectra) and ^{31}P MAS spectrum of neat, polycrystalline **dppbz** exposed to the atmosphere for 22 hours (bottom).

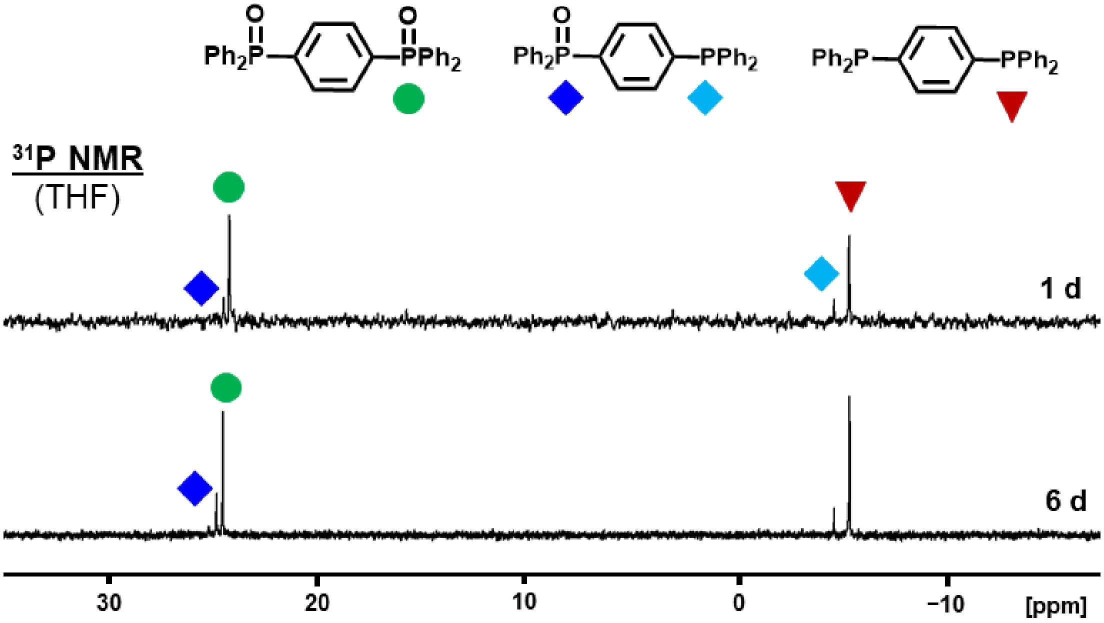

Figure S9. ^{31}P NMR spectra of **dppbz** adsorbed on AC with 92% surface coverage and exposed to air for 1 (top) and 6 days (bottom). The chemical shifts are 23.82 ppm for **dppbzO$_2$**, 24.06 and –4.64 ppm for **dppbzO**, and –5.34 ppm for **dppbz**.

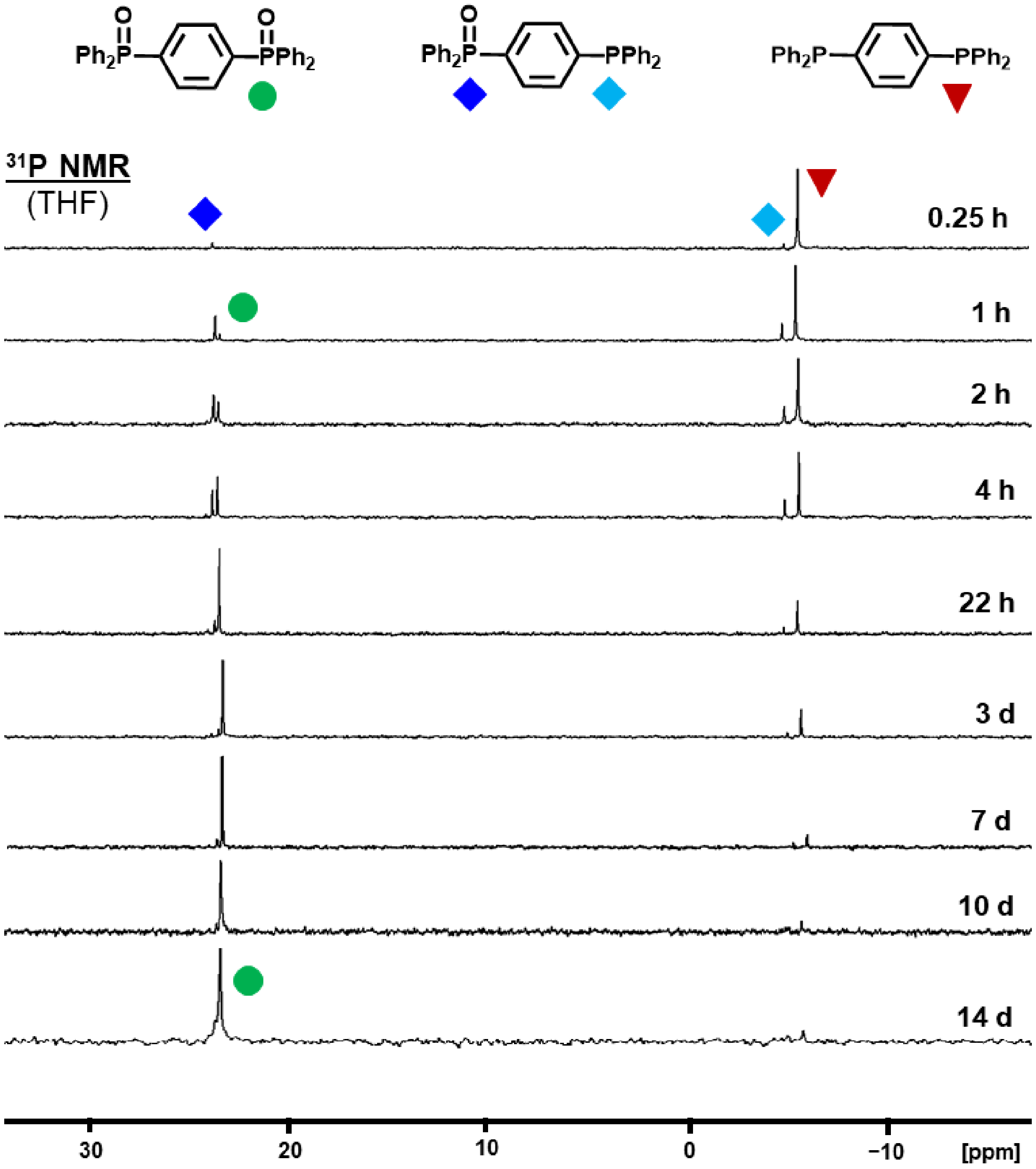

Figure S10. ^{31}P NMR spectra of **dppbz** adsorbed on AC with 40% surface coverage and measured after exposure to the atmosphere at the indicated times. The chemical shifts are 23.82 ppm for **dppbzO$_2$**, 24.06 and –4.64 ppm for **dppbzO**, and –5.34 ppm for **dppbz**.

Table S9. Presence of **dppbz**, the monoxide **dppbzO**, and **dppbzO$_2$** after the indicated times of air exposure of **dppbz** adsorbed on AC with 40% surface coverage (*92% surface coverage; exposure times one and six days). The values are based on the integration of the ^{31}P NMR signals.

**Time (h)**	**dppbz (%)**	**dppbzO (%)**	**dppbzO$_2$ (%)**
0.25	91	9	0
1	62	33	5
2	55	30	15
4.5	28	29	43
22	24	13	63
71.5	22	9	60
97	14	10	76
170	11	10	79
240	8	6	86
336	4	0	96
1 d*	35*	17*	48*
6 d*	38*	20*	42*

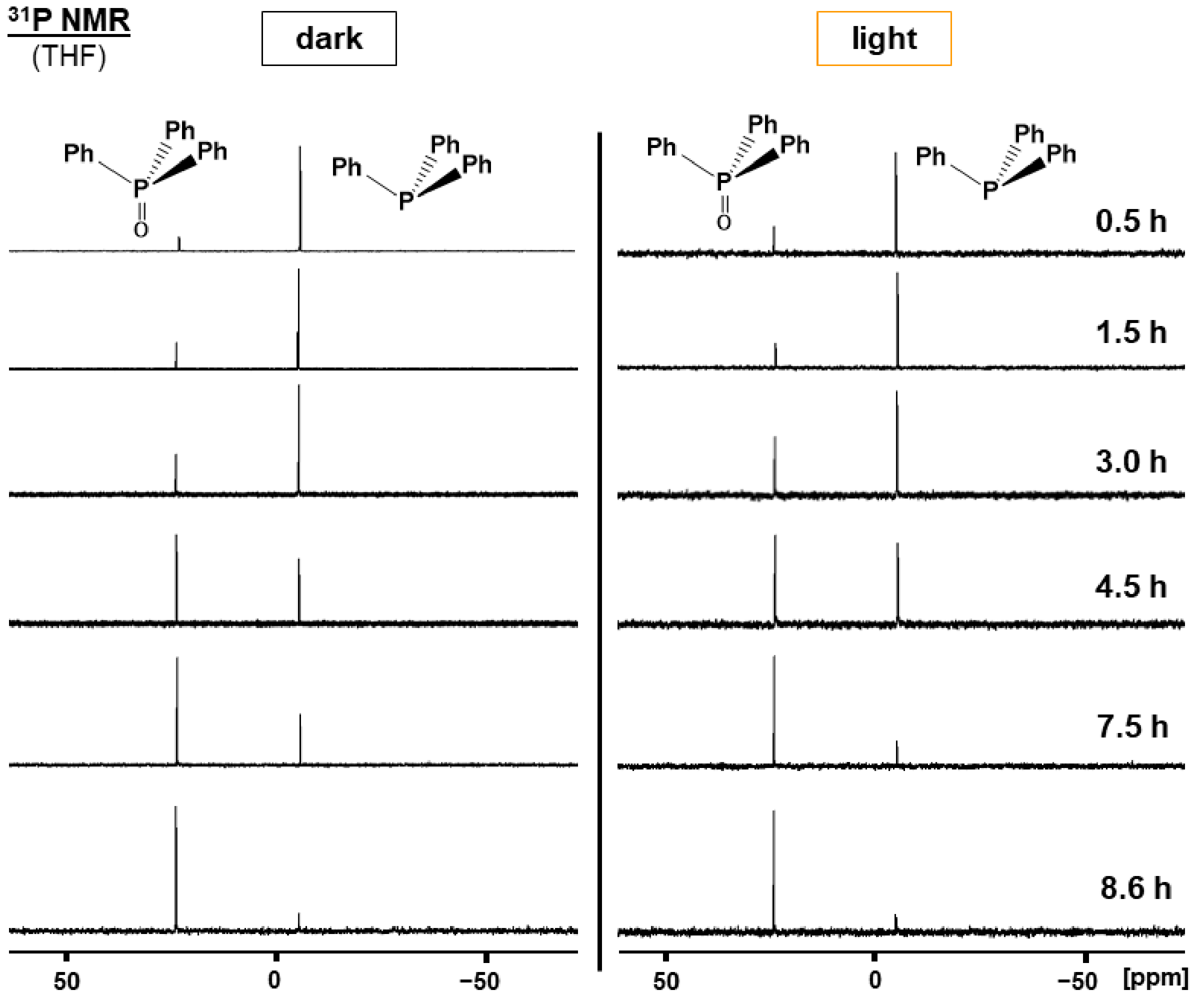

Figure S11. ^{31}P NMR spectra for monitoring the oxidation of PPh_3 adsorbed with 40% surface coverage on AC in the dark (left) and exposed to daylight (right). Samples were taken from both batches at the indicated times.

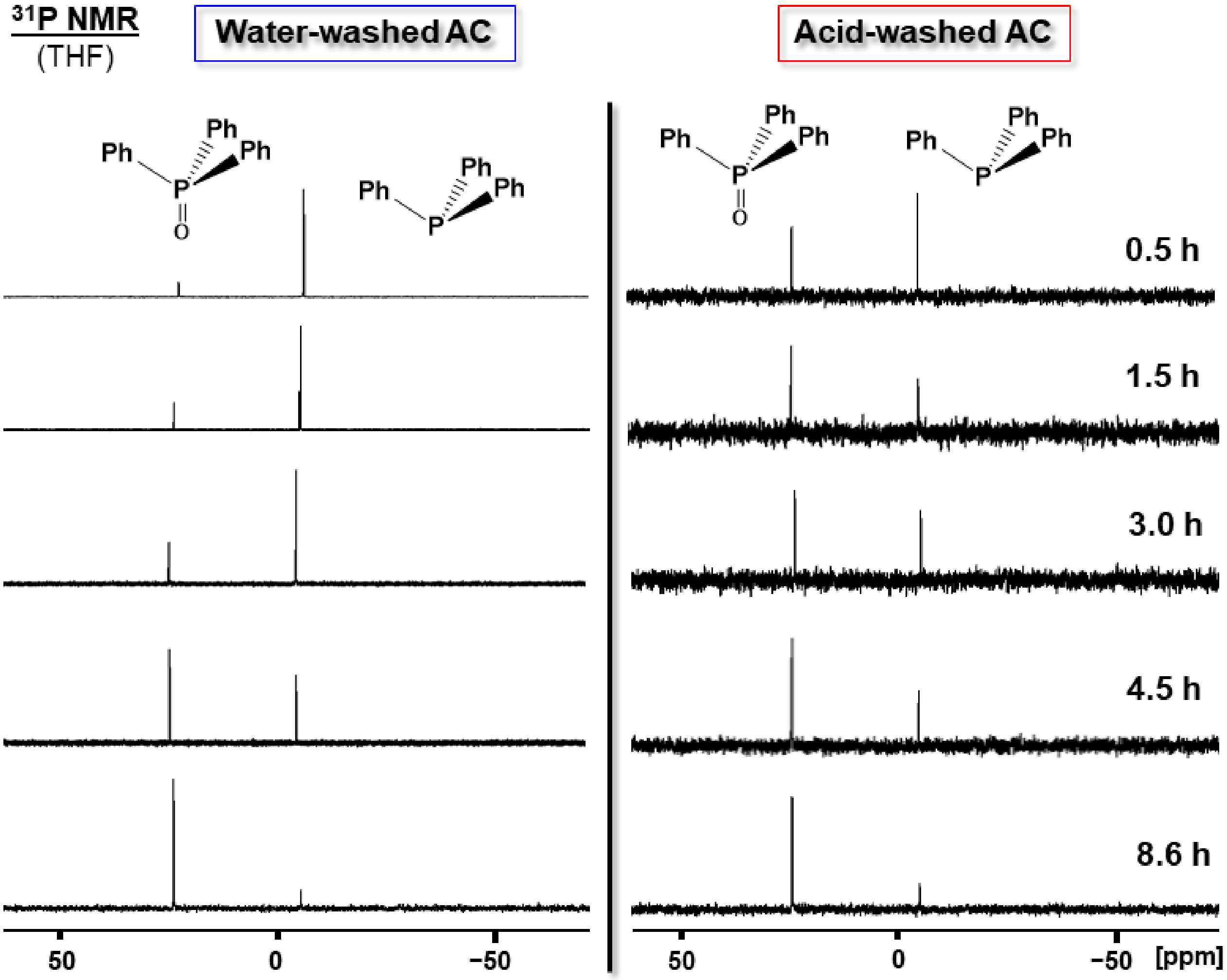

Figure S12. ^{31}P NMR spectra for monitoring the oxidation of PPh_3 adsorbed with 40% surface coverage on AC. The AC has been washed with water prior to use (left) and with aqueous HCl (right). Samples were taken from both batches at the indicated times.

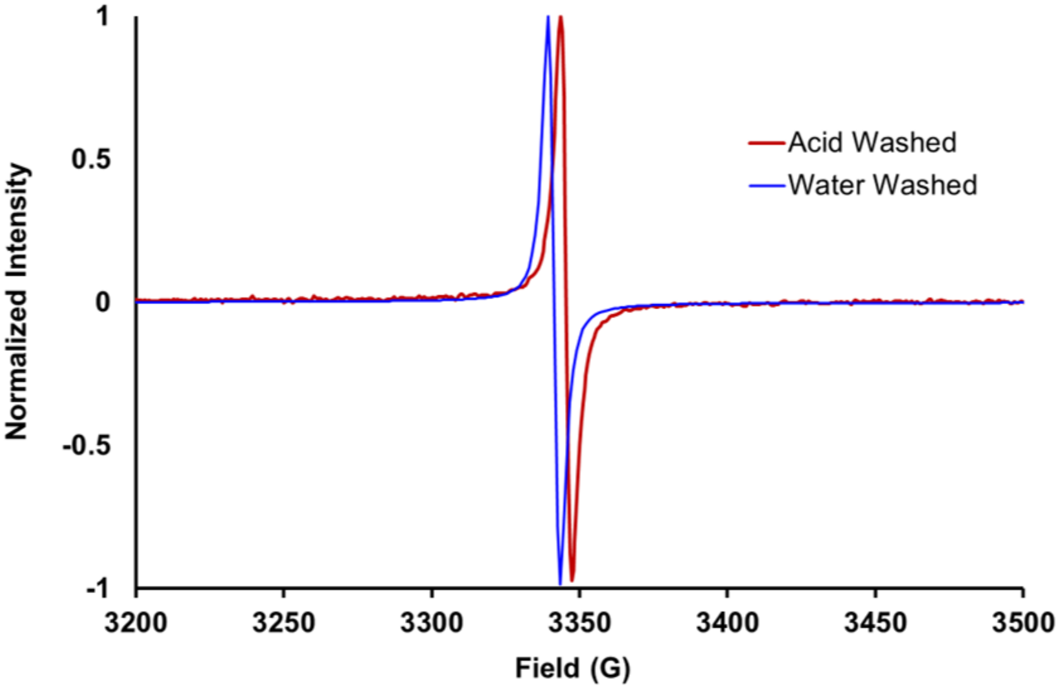

Figure S13. EPR spectra of AC after washing with HCl (red line) and with water (blue line).

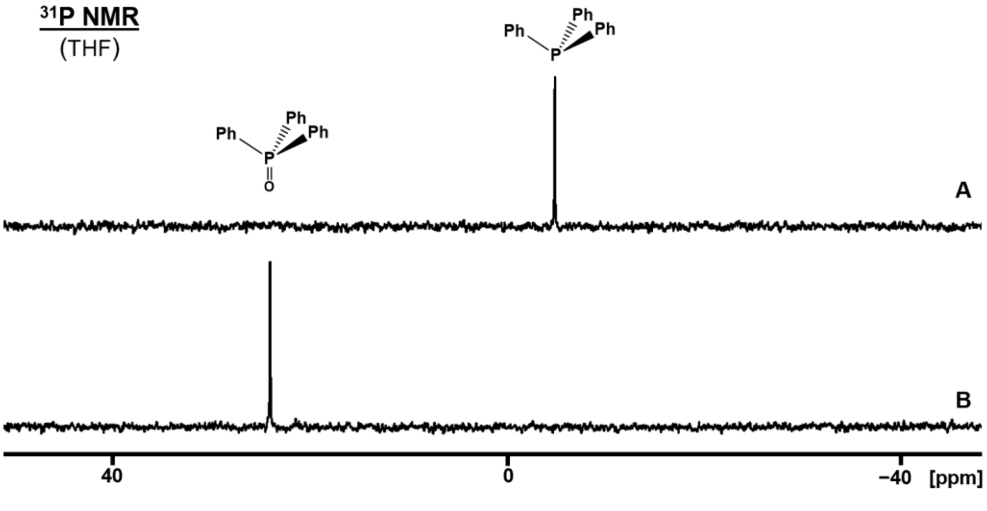

Figure S14. ^{31}P NMR spectra of a solution of PPh_3 in THF stirred at 65 °C for four hours under the atmosphere (**A**) and the same solution after adding 10 mol% AIBN and stirring for four hours under identical conditions (**B**).

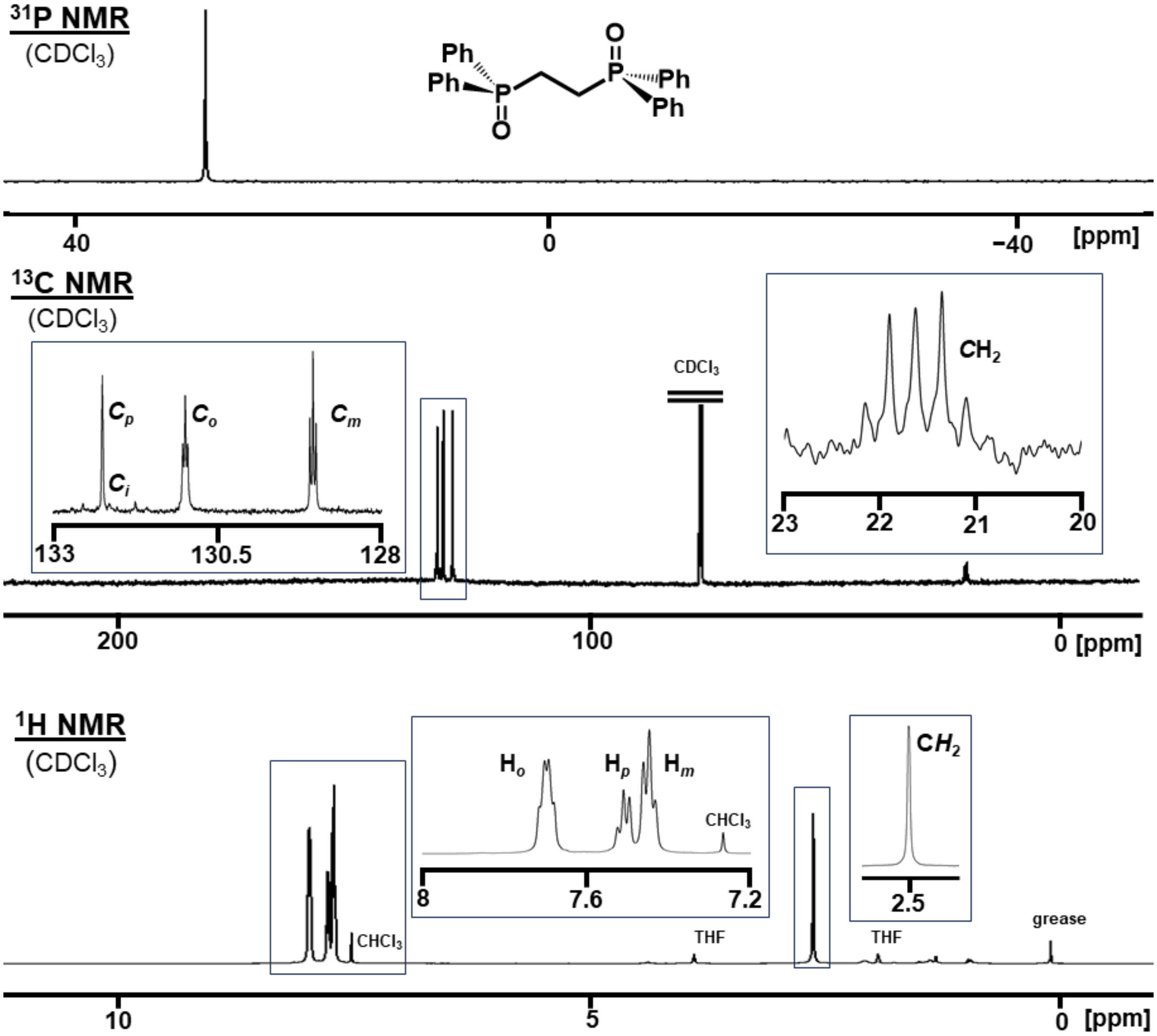

Figure S15. ^{31}P (top), ^{13}C (middle), and ^{1}H (bottom) NMR spectra of **dppeO₂** obtained quantitatively after adsorption on AC with 40% surface coverage and exposure to the atmosphere. All ^{13}C NMR signals, except that of C_p show virtual couplings.
